# Catecholamine treatment induces reversible heart injury and cardiomyocyte gene expression

**DOI:** 10.1186/s40635-024-00632-9

**Published:** 2024-05-11

**Authors:** Christine Bode, Sebastian Preissl, Lutz Hein, Achim Lother

**Affiliations:** 1 Institute of Experimental and Clinical Pharmacology and Toxicology, Faculty of Medicine, University of Freiburg, Freiburg, Germany; 2 BIOSS Centre for Biological Signaling Studies, University of Freiburg, Freiburg, Germany; 3 Interdisciplinary Medical Intensive Care, Medical Center-University of Freiburg, Faculty of Medicine, University of Freiburg, Freiburg, Germany

**Keywords:** Adrenergic receptors, Endothelin, Cardiomyocyte, Gene expression, Intensive care medicine

## Abstract

**Background:**

Catecholamines are commonly used as therapeutic drugs in intensive care medicine to maintain sufficient organ perfusion during shock. However, excessive or sustained adrenergic activation drives detrimental cardiac remodeling and may lead to heart failure. Whether catecholamine treatment in absence of heart failure causes persistent cardiac injury, is uncertain. In this experimental study, we assessed the course of cardiac remodeling and recovery during and after prolonged catecholamine treatment and investigated the molecular mechanisms involved.

**Results:**

C57BL/6N wild-type mice were assigned to 14 days catecholamine treatment with isoprenaline and phenylephrine (IsoPE), treatment with IsoPE and subsequent recovery, or healthy control groups. IsoPE improved left ventricular contractility but caused substantial cardiac fibrosis and hypertrophy. However, after discontinuation of catecholamine treatment, these alterations were largely reversible. To uncover the molecular mechanisms involved, we performed RNA sequencing from isolated cardiomyocyte nuclei. IsoPE treatment resulted in a transient upregulation of genes related to extracellular matrix formation and transforming growth factor signaling. While components of adrenergic receptor signaling were downregulated during catecholamine treatment, we observed an upregulation of endothelin-1 and its receptors in cardiomyocytes, indicating crosstalk between both signaling pathways. To follow this finding, we treated mice with endothelin-1. Compared to IsoPE, treatment with endothelin-1 induced minor but longer lasting changes in cardiomyocyte gene expression. DNA methylation-guided analysis of enhancer regions identified immediate early transcription factors such as AP-1 family members Jun and Fos as key drivers of pathological gene expression following catecholamine treatment.

**Conclusions:**

The results from this study show that prolonged catecholamine exposure induces adverse cardiac remodeling and gene expression before the onset of left ventricular dysfunction which has implications for clinical practice. The observed changes depend on the type of stimulus and are largely reversible after discontinuation of catecholamine treatment. Crosstalk with endothelin signaling and the downstream transcription factors identified in this study provide new opportunities for more targeted therapeutic approaches that may help to separate desired from undesired effects of catecholamine treatment.

**Supplementary Information:**

The online version contains supplementary material available at 10.1186/s40635-024-00632-9.

## Background

Catecholamines such as adrenaline (epinephrine) and noradrenaline (norepinephrine) are commonly used as therapeutic drugs in intensive care medicine to support organ function, e.g., in patients with cardiogenic or non-cardiogenic shock [1–4]. They are key regulators of blood pressure, heart rate, and cardiac contractility. In physiology, activation of adrenergic receptors represents an important short-term compensatory mechanism to maintain sufficient organ perfusion during increased demand due to stress, illness, or injury [5]. However, excessive or sustained activation drives detrimental cardiac remodeling, including cardiac hypertrophy, fibrosis, and inflammation, and may lead to heart failure [4–6]. Therapeutic catecholamine use is associated with an increased risk for arrhythmia and may cause myocardial necrosis [7, 8]. There are warnings that catecholamine treatment—in particular of substances with positive chronotropic effects—may increase mortality [1].

Adrenaline and noradrenaline are predominantly released by the adrenal glands and sympathetic nerve endings, respectively, and act via G-protein coupled α- and β-adrenergic receptors on numerous cell types [4, 5]. Ventricular cardiomyocytes predominantly express β_1_-, α_1A_-, and α_1B_-adrenergic receptors [9, 10]. Transgenic overexpression of β_1_-adrenergic receptors alters calcium handling in cardiomyocytes via activation of protein kinase A signaling and thereby increases contractility in young mice while persistent overactivation during aging leads to cardiomyocyte hypertrophy [11, 12]. Prolonged pharmacological stimulation of adrenergic receptors using combined treatment with α_1_- and β-adrenoreceptor agonists (in mice usually phenylephrine and isoprenaline) leads to heart failure [13–15]. This is associated with changes in gene expression related to extracellular matrix remodeling and inflammation [15].

Whether transient activation of the adrenergic system causes persistent myocardial injury remains debated: Takotsubo cardiomyopathy, a clinical syndrome that is associated with overactivation of the adrenergic system due to emotional stress or endocrine disorders such as pheochromocytoma, is typically characterized by transient left ventricular dysfunction with spontaneous recovery [16]. However, more recent data indicate that long-term outcome in patients with Takotsubo syndrome is comparable to that of acute coronary syndromes [17]. In mice, single high-dose application of isoprenaline induces reversible cardiomyocyte damage [8]. On the other hand, delayed upregulation of atrial natriuretic peptide precursor A (*Nppa)* gene expression in heart tissue only after isoprenaline withdrawal has been reported [18]. This implies that patients receiving catecholamine treatment may be at risk to develop secondary heart failure.

In this study, we assessed whether cardiac remodeling and pathological gene expression in cardiomyocytes induced by prolonged catecholamine exposure are reversible and identified molecular mechanisms involved in that process.

## Methods

### Agonist treatment

**Fig. 1:**
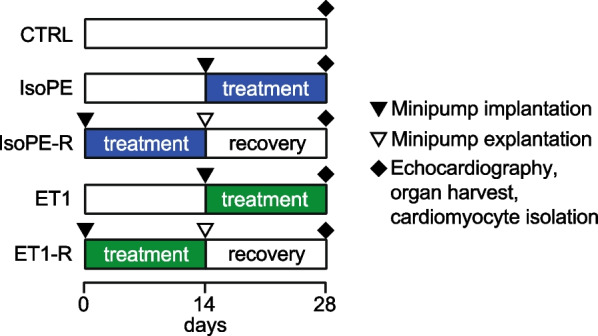
Study protocol. Adult male mice (*n* = 6 per group) were treated with isoprenaline and phenylephrine (IsoPE, blue) or endothelin-1 (ET1, green) from osmotic minipumps for 14 days. In half of the mice, minipumps were removed and the mice were allowed to recover for another 14 days (IsoPE-R; ET1-R). Implantation of minipumps in the IsoPE and ET1 groups was delayed to ensure that the animals were the same age at the end of the study period. Untreated mice served as controls (CTRL). Black triangle, minipump implantation; open triangle, minipump explantation; diamond, echocardiography, organ harvest and cardiomyocyte isolation

All animal experiments were carried out according to the European Community of guiding principles of care and use of animals (2010/63/EU). Authorizations were obtained from Regierungspräsidium Freiburg, Germany (G-16/62). The mice used in this study were 9–13-week-old C57BL/6N mice (Charles River Laboratories, RRID:MGI:2159965, *n* = 6 per group). Previous studies of isoproterenol-induced heart injury showed no difference between sexes [14], thus in agreement with 3R regulations we limited experiments to male mice. All animals were given 1 week of acclimatization at the facility and kept with undisturbed social interaction in individually ventilated cages under a 12 h light/dark cycle and ad libitum access to tap water and standard diet. Mice were assigned to 14 days treatment with isoprenaline and phenylephrine (IsoPE, 15 mg/kg body weight/d each), treatment with IsoPE and subsequent recovery (IsoPE-R), or healthy control (CTRL) groups. Following the findings from these experiments, we assigned additional mice to treatment with endothelin-1 (ET1, 36 µg/kg body weight/d) or treatment with ET1 and subsequent recovery (ET1-R). In the recovery groups, minipumps were removed after 14 days and the mice were allowed to recover for another 14 days. Treatment of mice assigned to the IsoPE and ET1 groups started after 14 days to ensure age matching with the CTRL and recovery groups at the end of the experiment (Fig. 1). Isoprenaline and phenylephrine [Sigma-Aldrich, dissolved in 0.9% NaCl and 0.1% ascorbic acid (Roth)] and endothelin-1 (Enzo Life Sciences, dissolved in 5% acetic acid) and delivered from subcutaneously implanted osmotic minipumps (Alzet, model 1002). For minipump implantation, anesthesia with isoflurane (1–2% v/v) and perioperative analgesia with carprofen was applied. At the end of the study period, the mice underwent echocardiography before being euthanized by cervical dislocation under deepened isoflurane anesthesia and hearts were collected for further analysis.

From here on, we use ‘ET1’ for endothelin-1 treatment, ‘*Edn1*’ for the endothelin-1 gene, and ‘endothelin-1’ for the endogenous peptide.

### Echocardiography

For echocardiography, mice were placed in supine position and kept under temperature- and ECG-controlled anesthesia (isoflurane 1–2% v/v). Imaging was performed on a Vevo 2100 System (FUJIFILM VisualSonics Inc.) equipped with an MS-400 transducer. B-Mode and M-Mode images were recorded in parasternal short and long axis. Left ventricular systolic and diastolic anterior and posterior wall thickness (LVAWs/d, LVPWs/d), interventricular septum thickness (IVSs/d), internal diameter (LVIDs/d), ejection fraction, fractional shortening, and stroke volume were analyzed using VevoLAB software V3.2.5 (FUJIFILM VisualSonics Inc.) and averaging 5 consecutive contraction cycles. Cardiac index was determined as the ratio of cardiac output and body weight. Heart rate was determined from the simultaneous ECG recordings using the Vevo 2100 System.

### Histology

Heart samples were fixed in 4% (m/v) paraformaldehyde, embedded in paraffin, and cut into 5 µm transversal sections. LV collagen deposition was determined from the ratio of picrosirius red positive area and total area using ImageJ software. LV cardiomyocyte cross sectional area was quantified by morphometry in sections stained with wheat germ agglutinin (Alexa488 conjugate, Invitrogen) and DAPI. Investigators performing histological analysis have been blinded for group allocation.

### qRT-PCR

LV samples were triturated with a TissueLyser LT system (Qiagen) and total RNA was extracted using AllPrep DNA/RNA Micro Kit (Qiagen) and transcribed into cDNA (QuantiTect Reverse Transcription Kit, Qiagen). qRT-PCR was performed with SYBR Green (Bio-Rad) and analyzed based on ∆∆CT calculations with *Rps29* as reference gene (Suppl. Fig. S1). Primer sequences are provided in Suppl. Table S1.

### Isolation of cardiomyocyte nuclei

Cardiomyocyte nuclei were isolated from frozen LV tissue as previously described [19]. Briefly, samples were transferred to ice cold lysis buffer and triturated using a gentleMACS dissociator. Cardiomyocyte nuclei were labelled using antibodies against pericentriolar material 1 (anti-PCM1, Sigma-Aldrich #HPA023374, RRID:AB_1855073) and phospholamban (PLN, Badrilla #A010-14, RRID:AB_2617049), and DRAQ7 (BD Biosciences #564904, RRID:AB_2869621) as a nuclear stain. PCM-1 and PLN positive nuclei were isolated using a BD FACS Melody cell sorter and lysed in buffer RLT Plus (Qiagen). RNA was isolated using AllPrep DNA/RNA Micro Kit (Qiagen).

### RNA-sequencing (RNA-seq)

RNA-seq libraries were prepared with the Ovation SoLo RNA-Seq Library Preparation Kit (NuGEN) from 10 ng of isolated RNA according to the manufacturer's instructions. Purification and size selection was carried out with Agencourt AMPure XP Beads (Beckman Coulter). DNA concentration was measured with Qubit dsDNA HS (high sensitivity) assay (Life Technologies). Fragment size and distribution was determined using the 2100 Bioanalyzer and High Sensitivity DNA Kit (Agilent). Sequencing was performed on a NovaSeq 6000 sequencer (50 base pair, Illumina) at the Max Planck Institute for Immunobiology and Epigenetics, Freiburg. Raw data is available via BioProject ID PRJNA1027750. Endothelial cell data was reanalyzed from a previously published data set [20], available via BioProject ID PRJNA945592.

### Bioinformatics

RNA sequencing data were analyzed using Galaxy (https://usegalaxy.eu) [21]. First 5 bp from the 5′ end of forward reads were trimmed using TrimGalore! to remove overhang of the forward adaptor and reads were mapped with RNA–STAR [22] to the mm10 mouse reference genome. Duplicates were removed by RmDup. mRNA expression was quantified by htseq-count [23]. Differential gene expression (*q* < 0.05, | fold change > 2) between groups was determined using DESeq2 [24] with *p* value adjusted for multiple testing by Benjamini–Hochberg correction and *n* = 6 samples per group. Genes that were differentially expressed after IsoPE were classified as recovered (IsoPE-R vs. IsoPE *q* < 0.05 and IsoPE-R vs. CTRL *q* > 0.05), partially recovered [(IsoPE-R vs. IsoPE and IsoPE-R vs. CTRL *q* > 0.05) or (IsoPE-R vs. IsoPE and IsoPE-R vs. CTRL *q* < 0.05)], or persistently regulated (IsoPE-R vs. IsoPE *q* > 0.05 and IsoPE-R vs. CTRL *q* < 0.05) after recovery.

GraphPad Prism 9.5.1, gplots heatmap2, and pyGenomeTracks [25] were used to visualize data. Functional enrichment analysis of biological processes from Gene Ontology (GO) database was carried out using Cytoscape with ClueGO [26].

Regulatory regions in cardiomyocytes identified by DNA methylation-guided annotation were derived from a previously published data set [27] and linked to gene expression using GREAT [28] version 4.0.4 (association rule: basal + extension: 5000 bp upstream, 1000 bp downstream, 1,000,000 bp max extension, curated regulatory domains). Enrichment of transcription factor binding motifs was analyzed using HOMER [29] version 4.11, tool *findMotifsGenome.pl* with option-size given.

### Statistical analysis

Statistical data analyses were performed with GraphPad prism 9.5.1. Results are presented as mean ± standard deviation (SD). If not stated otherwise, data were analyzed using One-way ANOVA followed by Bonferroni multiple comparison testing. *p* < 0.05 was considered statistically significant.

## Results

### Adrenergic stimulation induces hypercontractile left ventricular function and structural remodeling

**Fig. 2:**
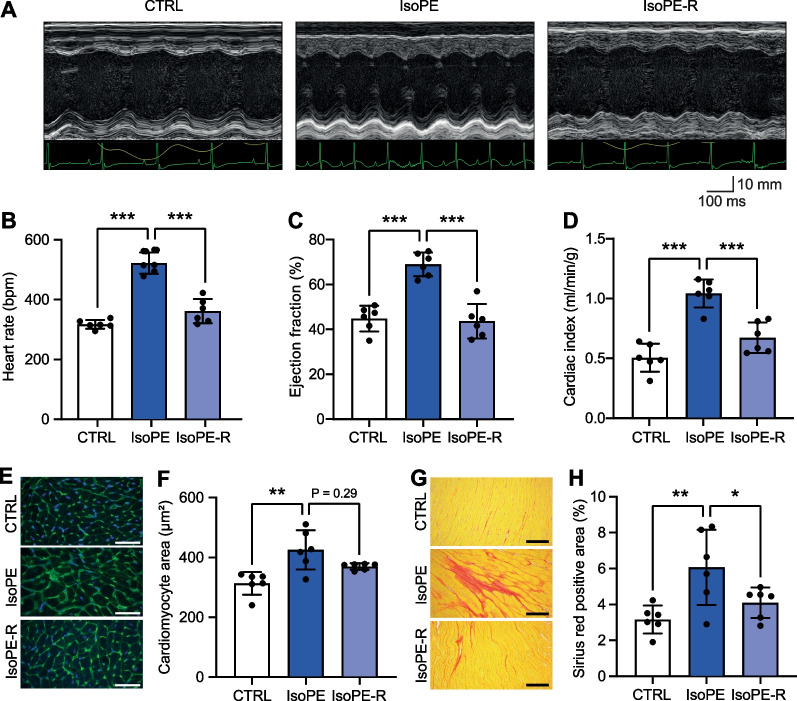
Left ventricular function and remodeling following catecholamine exposure.** A**, **B** Cardiac function was assessed by echocardiography (**A**, representative M mode recordings from parasternal short axis view) to determine heart rate (**B**), left ventricular ejection fraction (**C**), and cardiac index (**D**). **E, F**, Cardiomyocyte cross-sectional areas were determined by morphometry from left ventricular cross sections stained with wheat germ agglutinin. **G**, **H**, Interstitial fibrosis was determined by morphometry from left ventricular cross sections stained with picrosirius red. *n* = 6 per group. One-way ANOVA including ET1 and ET1-R followed by Bonferroni multiple comparison test (Table 1), *p < 0.05; **p < 0.01; ***p < 0.001

Left ventricular function after IsoPE treatment and after recovery was assessed by echocardiography (Fig. 2A–D, Table 1). As expected, IsoPE treated mice showed an increased heart rate (IsoPE 522 ± 36 bpm vs. CTRL 317 ± 15 bpm, *p* < 0.001, Fig. 2B) and left ventricular ejection fraction (IsoPE 69 ± 5% vs. CTRL 45 ± 6%, *p* < 0.001, Fig. 2C), resulting in a greater cardiac index (Fig. 2D). Within 14 days after removal of the minipump, cardiac function returned to baseline with no statistically significant difference between IsoPE-R and CTRL (Fig. 2B–D).

**Table 1:** Left ventricular structure and function after IsoPE or ET1 treatment

	CTRL	IsoPE	IsoPE-R	ET1	ET1-R
Heart rate (bpm)	317 ± 15	522 ± 36***	361 ± 40^###^	404 ± 16***	351 ± 10^§^
Fractional shortening (%)	22 ± 3	38 ± 4***	21 ± 5^###^	33 ± 5***	28 ± 2
Stroke volume (µl)	43 ± 10	54 ± 5	52 ± 5	56 ± 6*	59 ± 7^§§^
LV anterior wall thickness in diastole (mm)	0.72 ± 0.06	0.90 ± 0.11*	0.82 ± 0.14	0.82 ± 0.07	0.75 ± 0.07
LV anterior wall thickness in systole (mm)	0.97 ± 0.12	1.39 ± 0.17***	1.11 ± 0.19^##^	1.22 ± 0.07*	1.10 ± 0.05
LV internal diameter in diastole (mm)	4.26 ± 0.22	3.80 ± 0.31*	4.30 ± 0.25^#^	3.87 ± 0.24	4.16 ± 0.16
LV internal diameter in systole (mm)	3.33 ± 0.22	2.36 ± 0.30***	3.38 ± 0.31^###^	2.62 ± 0.31***	3.00 ± 0.18
LV posterior wall thickness in diastole (mm)	0.66 ± 0.14	0.81 ± 0.12	0.77 ± 0.18	0.70 ± 0.07	0.67 ± 0.06
LV posterior wall thickness in systole (mm)	0.91 ± 0.20	1.38 ± 0.21**	1.01 ± 0.24^#^	1.13 ± 0.19	1.01 ± 0.11
Heart weight / tibia length (mg/mm)	6.1 ± 0.4	7.3 ± 0.3***	6.4 ± 0.2^###^	6.6 ± 0.3*	6.7 ± 0.3**
Sirius red positive area (%)	3.1 ± 0.8	6.0 ± 2.1**	4.1 ± 0.8^#^	3.1 ± 0.8	3.1 ± 0.4

*n* = 6 per group, mean ± SD. One-way ANOVA followed by Bonferroni multiple comparison test. *p < 0.05, **p < 0.01, ***p < 0.001 vs. CTRL; ^#^*p* < 0.05, ^##^*p* < 0.01, ^###^*p* < 0.001 vs. IsoPE; ^§^*p* < 0.05, ^§§^*p* < 0.01, ^§§§^*p* < 0.001 vs. ET1

Despite a positive effect on LV function, IsoPE treatment caused adverse structural remodeling of the heart: Heart weight to tibia length ratio increased from 6.1 ± 0.4 mg/mm in untreated mice to 7.3 ± 0.3 mg/mm after IsoPE treatment (IsoPE vs. CTRL, *p* < 0.001), and returned to 6.4 ± 0.2 mg/mm after recovery (IsoPE-R vs. CTRL, *p* < 0.001). Cardiomyocyte cross-sectional area was 310 ± 38 µm^2^ in untreated mice, 422 ± 66 µm^2^ after IsoPE treatment, and 367 ± 11 µm^2^ after recovery (Fig. 2E, F). The extent of left ventricular interstitial fibrosis, as determined by the sirius red positive area, was 3.1 ± 0.8% in untreated mice, 6.0 ± 2.1% after IsoPE treatment, and 4.1 ± 0.8% after recovery (Fig. 2G, H).

### Gene expression in cardiomyocytes in response to catecholamine treatment

**Fig. 3:**
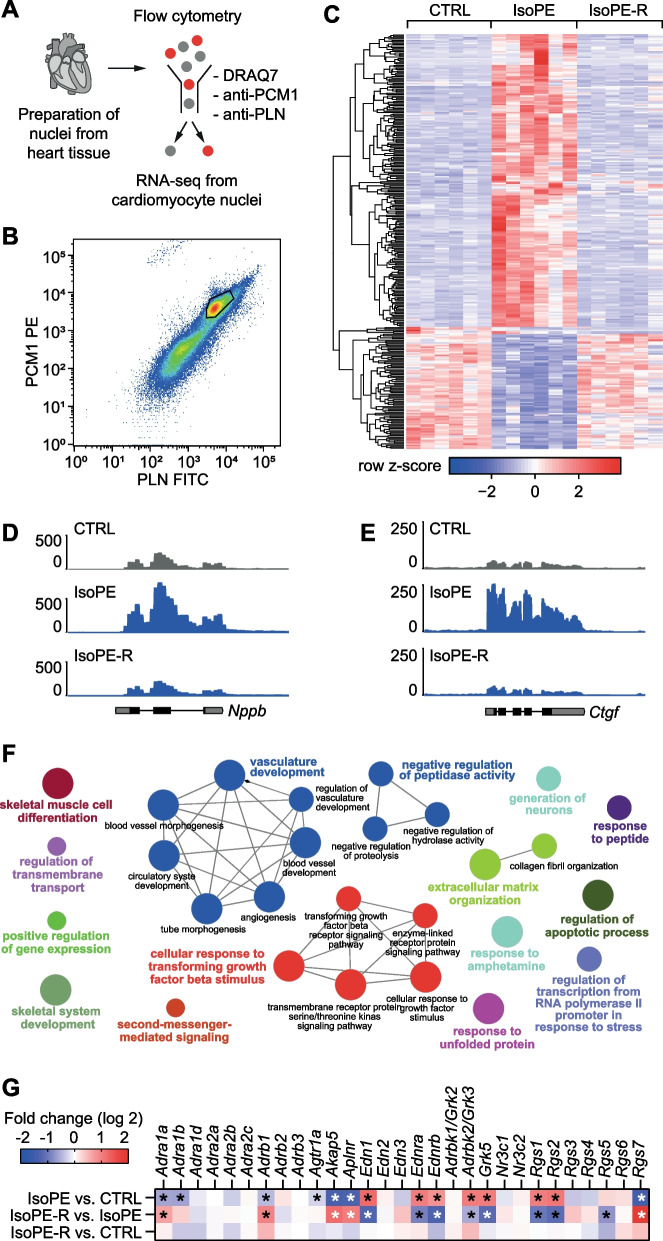
Gene expression in isolated cardiomyocyte nuclei after catecholamine exposure. **A**, **B**, Cardiomyocyte nuclei were isolated from heart tissue by fluorescence activated sorting using antibodies against pericentriolar material 1 (PCM1) and phospholamban (PLN) and DRAQ7 as a nuclear stain. **C** Heatmap representing genes that were differentially expressed (fold change > 2, *q* < 0.05) in cardiomyocyte nuclei after treatment with isoprenaline and phenylephrine or after recovery as determined by RNA-seq. **D**, **E** Representative traces showing mRNA expression at the natriuretic peptide precursor B (*Nppb*) or connective tissue growth factor (*Ctgf*) locus. **F** Biological processes enriched (*p* < 0.05) among genes that were differentially expressed after IsoPE treatment compared to CTRL as derived from gene ontology. **G**, mRNA expression of selected genes related to adrenergic signaling, endothelin-1 signaling, or renin–angiotensin–aldosterone system signaling pathways. *n* = 6 per group. *q < 0.05. CTRL, untreated control; IsoPE, mice treated with isoprenaline and phenylephrine; IsoPE-R, mice allowed to recover after treatment with isoprenaline and phenylephrine

The heart is composed of numerous cell types that show distinct gene expression profiles [30]. To assess the impact of catecholamine treatment on cardiomyocyte gene expression, mRNA expression in isolated cardiomyocyte nuclei was determined by RNA-seq (Fig. 3A, B). After IsoPE treatment, 295 genes were differentially expressed compared to untreated mice (210 upregulated, 85 downregulated; fold change > 2, *q* < 0.05; Fig. 3C, Suppl. Table S2). These included typical markers of cardiac remodeling such as atrial natriuretic peptide precursor B (*Nppb*, 2.4-fold up; Fig. 3D) or connective tissue growth factor (*Ctgf*, 6.5-fold up; Fig. 3E). Gene ontology analysis revealed an enrichment of genes related to biological processes such as cellular response to transforming growth factor stimulus, vasculature development, or extracellular matrix organization among the differentially expressed genes (Fig. 3F).

We analyzed the impact of IsoPE on the expression of adrenergic receptors and components of related signaling pathways in cardiomyocytes (Fig. 3G). The expression of the main receptors for isoprenaline and phenylephrine in cardiomyocytes, β_1_-adrenergic receptor (*Adrab1*, 1.4-fold down) and α_1_-adrenergic receptors (*Adra1a*, 1.5-fold down; *Adra1b*, 1.6-fold down), was moderately downregulated after IsoPE treatment. In addition, we observed several changes in related G-protein coupled receptor signaling (Fig. 3G). Interestingly, mRNA expression of endothelin-1 (*Edn1*, 2.4-fold up) and its receptors (*Ednra*, 2.0-fold up; *Ednrb*, 1.8-fold up) was induced in cardiomyocytes from IsoPE treated animals. All the before mentioned changes returned to baseline after recovery (Fig. 3G, Suppl. Table S2).

### Endothelin-1-induced gene expression in cardiomyocytes

**Fig. 4:**
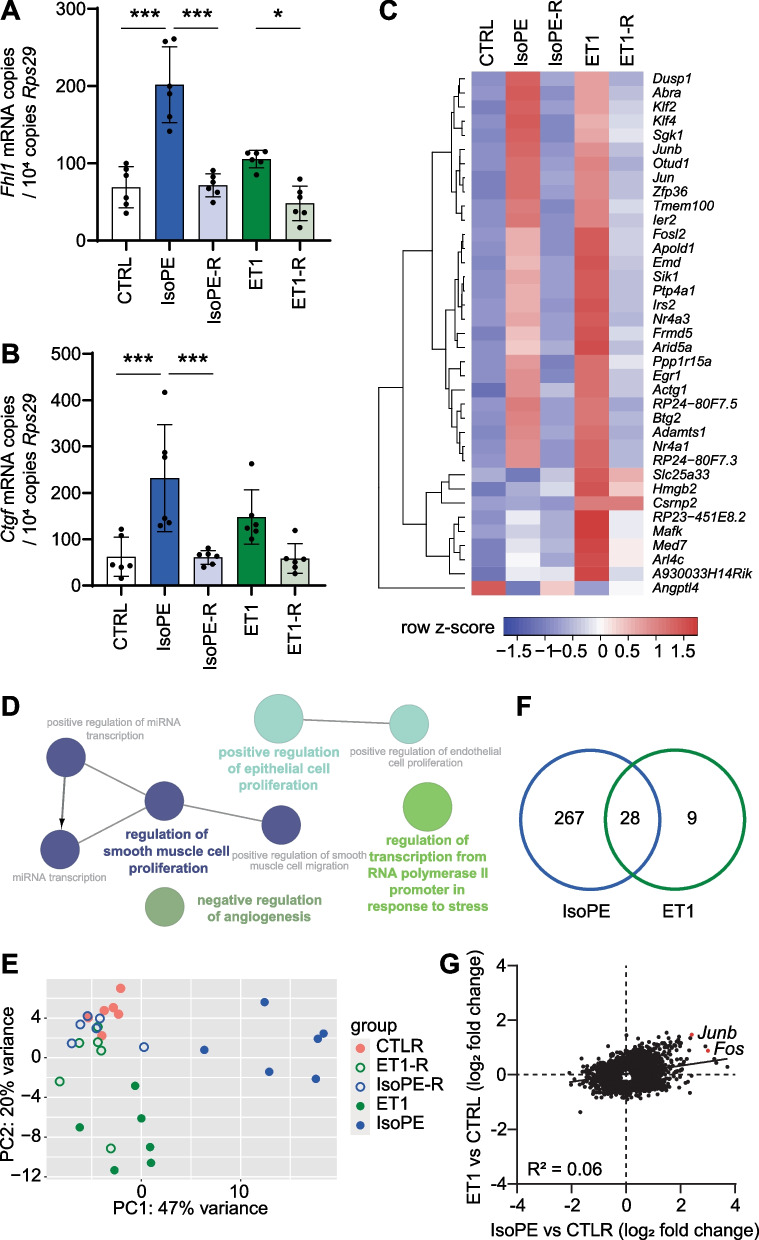
Gene expression response to ET1 compared to adrenergic stimulation. **A**, **B** mRNA expression of four-and-a-half LIM domains 1 (*Fhl1*) and connective tissue growth factor (*Ctgf*) in left ventricular tissue after treatment with isoprenaline and phenylephrine (IsoPE), IsoPE and recovery (IsoPE-R), endothelin-1 (ET1), or ET1 and recovery (ET-1R). *n* = 6 per group. One-way ANOVA followed by Bonferroni multiple comparison test, *p < 0.05; ***p < 0.001 (cross-comparisons of IsoPE vs. ET1 groups not shown). **C** Heatmap representing genes that were differentially expressed (fold change > 2, *q* < 0.05) in cardiomyocyte nuclei after ET1 treatment (*n* = 6 per group). **D** Biological processes enriched (*p* < 0.05) among genes that were differentially expressed after ET1 treatment compared to CTRL as derived from gene ontology. **E** Principal component analysis. **F** Venn diagram representing numbers of differentially expressed genes (fold change > 2 vs. CTRL, *q* < 0.05) after IsoPE, ET1, or both. **G** Correlation of gene expression changes after IsoPE or ET1 vs. CTRL (*q* < 0.05)

Endothelin-1 is a peptide hormone that acts on the heart and the vasculature [31]. We hypothesized that the upregulation of endothelin-1 may augment the effect of IsoPE on cardiomyocytes via autocrine or paracrine action. Thus, we compared the effect of IsoPE to 14 days ET1 infusion to identify overlapping gene expression responses. ET1 increased left ventricular ejection fraction (ET1 61 ± 7% vs. CTRL 45 ± 6%, *p* < 0.001) and heart weight to tibia length ratio (ET1 6.6 ± 0.3 mg/mm vs. CTRL 6.1 ± 0.4 mg/mm, *p* < 0.05) compared to untreated controls (Table 1). Expression of marker genes for hypertrophy or fibrosis in heart tissue was less evident than with IsoPE (Fig. 4A, B). In isolated cardiomyocyte nuclei we found 37 genes differentially expressed compared to untreated mice (fold change > 2, *q* < 0.05; Fig. 4C, Suppl. Table S2). These were predominantly associated with regulation of smooth muscle cell proliferation (Fig. 4D). Overall, gene expression following ET1 and IsoPE showed only a weak correlation (Fig. 4E–G). 28 of 37 genes showed overlapping regulation with ET1 and IsoPE (Fig. 4C, F). Among the genes that showed overlapping regulation were immediate early transcription factors *Jun*, *Junb*, *Fosl2*, and *Egr1* (Fig. 4C, G, Suppl. Table S2).

### Comparison of gene expression following adrenergic stimulation in cardiomyocytes and endothelial cells

Adrenergic stimulation may not only affect cardiomyocytes but also non-myocytes such as endothelial cells or the crosstalk of both. To compare IsoPE-induced gene expression in cardiomyocytes and cardiac endothelial cells, we reanalyzed a previously published data set [20]. We found 735 genes differentially expressed in cardiac endothelial cells after IsoPE compared to untreated mice (fold change > 2, *q* < 0.05; Suppl. Table S2), with 43 of them overlapping with cardiomyocytes (Suppl. Fig. S2A). The overlapping genes again included immediate early transcription factors (*Jun*, *Junb*, *Fos*, *Fosl2*, *Egr1*) and other transcription factors (*Atf3*, *Klf2*, *Klf4*, *Nr4a1*, *Nr4a2*, *Nr4a3*) (Suppl. Fig. S2B, C). Endothelial cells are an important source of endothelin-1 [31]; however, we did not observe differential expression of *Edn1*, *Ednra*, and *Ednrb* genes after IsoPE treatment (Suppl. Table S2).

### Regulation of catecholamine-induced gene expression

**Fig. 5:**
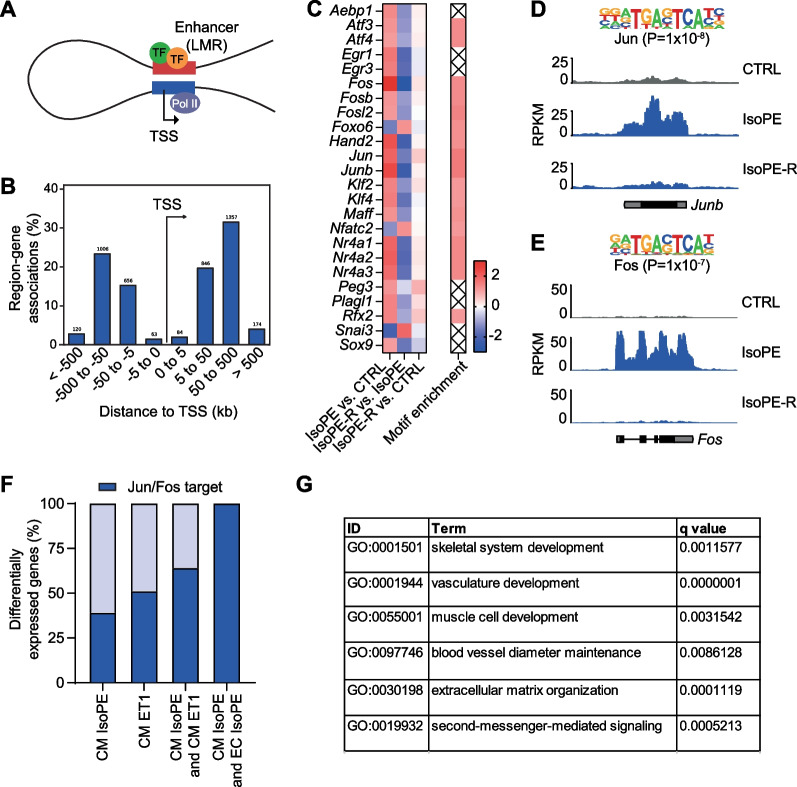
Regulators of gene expression response to adrenergic stimulation. **A** Genes that were differentially expressed after IsoPE treatment were linked to previously identified enhancer elements (low methylated regions, LMRs) in cardiomyocytes [27]. **B** Displayed is the relative position of regulatory elements to the respective transcription start site (TSS). **C** Heatmap showing mRNA expression (log_2_ fold change of transcription factors, defined as members of gene ontology term GO:0003700) and enrichment of their respective binding motifs within regulatory elements (fold change). Crossed-out elements indicate no statistically significant enrichment. **D**, **E** Motif enrichment and mRNA expression of AP-1 transcription factors Jun and Fos. **F** Proportions of Jun or Fos target genes among genes that were differentially expressed after IsoPE or ET1 treatment, the overlap of both, or among genes that were differentially expressed after IsoPE in cardiomyocytes (CM) and endothelial cells (EC). **G** Biological processes enriched (*p* < 0.05) among predicted Jun or Fos target genes that were differentially expressed (fold change > 2, *q* < 0.05) after IsoPE treatment as derived from gene ontology. TF, transcription factor; Pol II, RNA polymerase II

Gene expression is regulated by transcription factors binding to distal regulatory regions (enhancers) within the chromatin that physically interact with the promoter region of their target genes (Fig. 5A). We applied a previously published data set of regulatory regions in cardiomyocytes identified by DNA methylation-guided annotation [27] and linked these regions to IsoPE-induced differential gene expression (Fig. 5B). Genes that were differentially expressed after IsoPE treatment showed a strong enrichment of binding motifs for myocyte enhancer factor-2 (Mef2) and GATA family members in their associated regulatory regions (Suppl. File S1). We intersected the list of enriched binding motifs with mRNA expression of the respective transcription factors (Fig. 5C). Among the upregulated transcription factors with corresponding binding motif enriched were AP-1 family members Jun (*Jun*, 3.8-fold up, *Junb* 5.3-fold up) and Fos (*Fos*, 8.0-fold up; *Fosb* 2.2-fold up) (Fig. 5D, E). We predicted 115 genes among the 295 differentially expressed genes in IsoPE to be direct Jun or Fos target genes (38.9% compared to 1.6% of all detected genes) (Fig. 5F, Suppl. Table S2). Notably, the proportion of Jun or Fos target genes increased when considering genes with overlapping regulation in cardiomyocytes after either IsoPE or ET1 treatment (64.3%) or in cardiomyocytes and endothelial cells after IsoPE treatment (100%) (Fig. 5F). Gene ontology analysis linked direct Jun or Fos target genes in cardiomyocytes to biological processes that are related to cardiac remodeling (Fig. 5G), thus making relevant contribution to the overall effect of IsoPE.

### Reversibility of gene expression after withdrawal of catecholamines

**Fig. 6:**
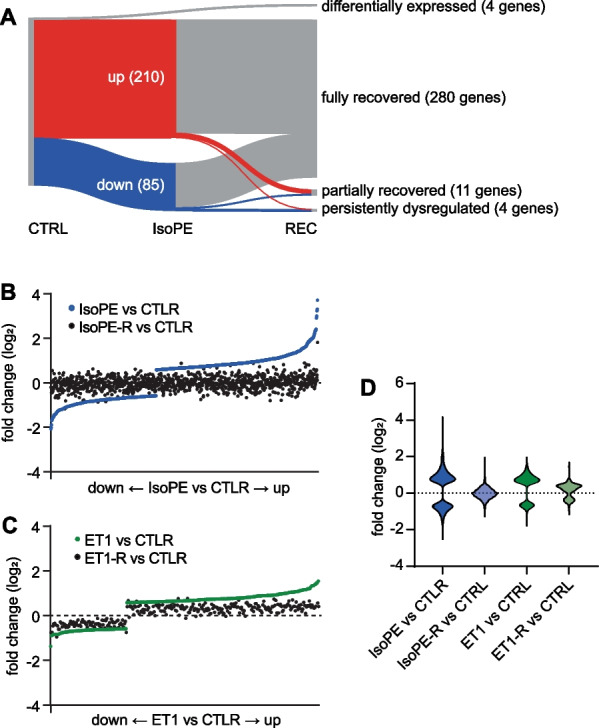
Reversibility of the gene expression response to adrenergic or ET1 stimulation. Sankey plot indicating genes that were fully recovered, partially recovered, persistently dysregulated, or differentially expressed after recovery from IsoPE as determined by RNA-seq of isolated cardiomyocyte nuclei (**A**). Genes that were differentially expressed (fold change > 1.5 vs. CTRL, *q* < 0.05) after IsoPE (**B**) or ET1 (**C**) treatment were ranked by magnitude of change. Waterfall plots (**B**, **C**) and violin plots (**D**) comparing differences in gene expression after IsoPE or recovery from IsoPE (IsoPE-R) and ET1 or recovery from ET1 (ET1-R) versus CTRL

After a 14 days recovery period, IsoPE-induced gene expression largely returned to baseline (Fig. 6A). 80 (94.9%) and 11 (3.7%) out of 295 differentially expressed genes showed full or partial recovery, respectively (Fig. 6A, Suppl. Table S2). Expression of immediate early transcription factors, including *Jun, Junb, Fos*, or *Fosl2,* returned to normal (Suppl. Table S2). Only 4 genes (1.4%) remained dysregulated after recovery (*Peg3*, *Cyp26b1*, *Gm38031*, *Pnpla3*) when compared to untreated controls. 4 genes (*Thbs1*, *Slc41a3*, *Arntl*, *Garnl3*) that have not been regulated in IsoPE were differentially expressed in recovered mice compared to control (Fig. 6A). Recovery was independent of the extent of gene regulation after IsoPE (IsoPE vs. CTRL and IsoPE-R vs. CTRL, slope 0.06, *R*^2^ = 0.04) (Fig. 6B). In contrast, although the extent of regulation was weaker overall, we observed a remaining offset after recovery from ET1 (Fig. 6C). Here, ET1 vs. CTRL and ET1-R vs. CTRL showed a stronger linear correlation (slope 0.49, *R*^2^ = 0.80). This was reflected by the mean absolute value of fold change (log_2_) vs. untreated control which was 0.22 ± 0.19 after recovery from IsoPE and 0.36 ± 0.17 after recovery from ET1 (Fig. 6D). In line with this, heart weight to tibia length ratio was persistently increased after withdrawal of ET1 (ET-R 6.7 ± 0.3 mg/mm vs. CTRL 6.1 ± 0.4 mg/mm, *p* < 0.05, Table 1).

## Discussion

**Fig. 7:**
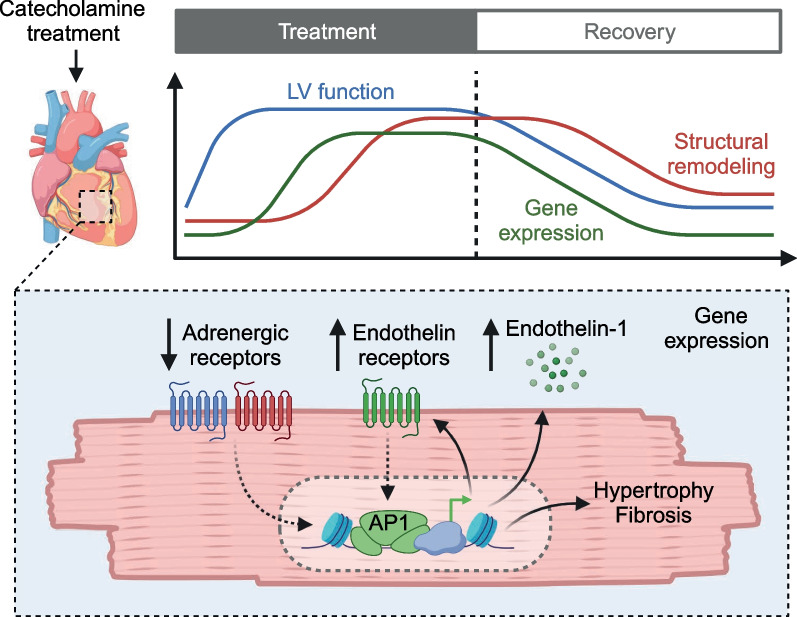
Summary. Estimated course of left ventricular function, structural remodeling, and cardiomyocyte gene expression during catecholamine treatment and after recovery. Key findings from RNA-seq of isolated cardiomyocyte nuclei after catecholamine treatment. LV: left ventricular. Created with BioRender.com

In this study, we investigated the time course of cardiac injury during and after catecholamine treatment and the molecular mechanisms involved in an experimental model. The four key findings of our study are that (1) catecholamine treatment induces structural cardiac damage before loss of function is detectable, (2) catecholamine exposure leads to downregulation of the β-adrenergic signaling pathway and activation of the endothelin signaling pathway in cardiomyocytes, (3) immediate early transcription factors are common regulators of the otherwise distinct pathological gene expression programs induced by adrenergic or endothelin-1 signaling, and (4) structural changes and gene expression recover after discontinuation of catecholamine treatment, while ET1 induces weaker but longer-lasting effects (Fig. 7).

The doses of isoprenaline and phenylephrine used in this study were suitable to maintain a hypercontractile state over a duration of 2 weeks, thus mimicking the therapeutic use of catecholamines in critically ill patients. Despite enhanced LV function under catecholamine treatment we observed the typical characteristics of adverse cardiac remodeling, such as interstitial fibrosis and cardiomyocyte hypertrophy. From a translational perspective, this suggests that subclinical myocardial injury may occur in patients treated with catecholamines before LV dysfunction becomes apparent. In line with this, elevated troponin T levels, indicating myocardial necrosis, have been observed in patients with sepsis or undergoing non-cardiac surgery and treated with catecholamines [32–34]. In our study, cardiac remodeling largely recovered after discontinuation of catecholamine treatment which is in line with a previous report using a similar model [35]. However, we cannot exclude that more severe injury may occur in patients who are elderly, have pre-existing cardiovascular disease, or increased levels of pro-fibrotic cytokines, e.g. in sepsis. Therefore, regular testing of cardiac biomarkers and echocardiography during catecholamine treatment and in long-term follow-up [36] should be considered. In addition, there is growing body of evidence that short acting beta blockers may improve outcomes of patients with septic shock [37] and a well-powered prospective trial is currently ongoing (clinicaltrials.gov NCT04748796).

To elucidate the underlying molecular mechanisms involved in that process, we studied the transcriptional response to IsoPE in cardiomyocytes. This approach allowed us to decipher the regulation of signaling pathways within a given cell type in a less biased manner compared to the analysis of cardiac tissue [27, 30, 38]. Studies comparing the effects of combined IsoPE treatment to isoprenaline alone indicated that concurrent activation of α-adrenergic receptors enhances the effect of isoprenaline on cardiac remodeling and gene expression [15, 35]. Sustained overactivation of β_1_-adrenergic receptors in heart failure results in desensitization of the receptor and its downstream signaling pathways, including downregulation of *Adrab1* gene expression and phosphorylation of the receptor [39, 40]. These mechanisms protect the heart against detrimental consequences, but also blunt the inotropic effects of catecholamine treatment [41]. We provide here an inventory of the transcriptional changes associated with β_1_-adrenergic receptor desensitization and show that gene expression is fully restored after withdrawal of the stimulus. Interestingly, we observed an upregulation of G-protein coupled receptor kinase GRK5 but not GRK2, which is considered to have a central role in β_1_-adrenergic receptor phosphorylation [40]. Although GRK5, like GRK2, desensitizes β_1_-adrenergic receptors, it induces cardiomyocyte hypertrophy by directly interacting with nuclear transcription factors [42]. RGS2, a known inhibitor of G-protein Gα_q_-mediated signaling that attenuates PE-induced cardiomyocyte hypertrophy [43, 44], was upregulated after IsoPE treatment. In addition to Gα_q_, RGS2 interacts with adenylyl cyclase and thereby blocks Gα_s_-dependent signaling downstream of β_1_-adrenergic receptors [44]. Modulation of the downstream signaling cascade may be a promising strategy to balance the desired and undesired effects of catecholamine treatment.

While components of the adrenergic receptor signaling pathway were downregulated during catecholamine treatment, we observed an upregulation of *Edn1*, *Ednra*, and *Ednrb* in cardiomyocytes. *Edn1* mRNA is translated into preproendothelin-1 which undergoes processing by endopeptidases and endothelin-converting enzyme into the active endothelin-1 peptide [31]. Endothelin-1 is produced by several cell types of the heart, including cardiomyocytes and endothelial cells, and promotes cardiomyocyte hypertrophy and fibrotic gene expression [31, 45–47]. In cultured cardiomyocytes, isoprenaline-induced upregulation of *Nppa* is prevented when processing into endothelin-1 by endothelin-converting enzyme or endothelin-1 secretion is inhibited [48]. This supports the conclusion that adrenergic receptor activation increases endothelin-1 secretion from cardiomyocytes, which may then act in an autocrine or paracrine manner to promote hypertrophic gene programs.

In our model, treatment with ET1 had only mild impact on cardiac function and remodeling, and induced a largely distinct gene expression program when compared to IsoPE. However, we identified immediate early transcription factors such as AP-1 family members Jun and Fos as key transcriptional regulators of the overlapping gene expression response to adrenergic and ET1 induced signaling in cardiomyocytes. AP-1 family members typically bind to distal enhancer regions rather than the transcription start site to control gene expression [49] and upregulation of AP-1 transcription factors in response to adrenergic stimulation has been reported before [35, 50]. In this study, we were able to intersect transcription factor expression with motif enrichment at cardiomyocyte enhancers and could thereby link Jun or Fos to their target genes, including pro-fibrotic factors such as connective tissue growth factor, fibronectin 1, or matrix metalloproteinase 2 [51–53]. In addition, we found that Jun and Fos target their own genes and the *Edn1* gene, pointing to a feed-forward mechanism. Epigenetic silencing of respective transcription factor binding sites is an emerging approach for targeted modulation of pathological gene expression in cardiomyocytes that may help to prevent adverse effects of catecholamine treatment [54].

After discontinuation of catecholamine treatment, cardiac remodeling and gene expression largely returned to baseline. We did not find specific gene programs to be activated during the recovery period, suggesting that cardiomyocytes are drivers of fibrosis progression while its resolution is rather mediated by other cell types, e.g. macrophages [55]. Although the extent of regulation in response to ET1 was weaker than with IsoPE, gene expression changes induced by ET1 incompletely recovered after minipump removal. This finding is consistent with a previous report describing a long-lasting hypertrophic response of cardiomyocytes after short-term ET1 stimulation in vitro that was associated with sustained MAPK signaling and insensitive to antagonist treatment [46]. Thus, reverse remodeling after injury seems independent of specific gene programs but rather depends on the type of ligand.

In conclusion, the results from this study show that prolonged catecholamine exposure induces adverse cardiac remodeling and gene expression before the onset of left ventricular dysfunction which has implications for clinical practice. The observed changes depend on the type of stimulus and are largely reversible after discontinuation of catecholamine treatment. Crosstalk with endothelin-1 signaling and the downstream transcription factors identified in this study provide new opportunities for more targeted therapeutic approaches that may help to separate desired from undesired effects of catecholamine treatment.

### Supplementary Information

Supplementary Material 1.

Supplementary Material 2.

Supplementary Material 3.

## Data Availability

Raw data are available via BioProject ID PRJNA1027750.

## Abbreviations

*Edn1*: Endothelin-1 gene
ET1: Treatment with endothelin-1
ET1-R: Treatment with ET1 and subsequent recovery
IsoPE: Treatment with isoprenaline and phenylephrine
IsoPE-R: Treatment with IsoPE and subsequent recovery
GO: Gene ontology
GRK: G-protein coupled receptor kinase
