# Supplementary material for: Catecholamine treatment induces reversible heart injury and cardiomyocyte gene expression
Source: Intensive Care Med Exp. 2024 May 11;12:48. doi: 10.1186/s40635-024-00632-9 (PMC11088585; doi:10.1186/s40635-024-00632-9)
Supplement: Supplementary file 2 — Supplementary Material 2. [file 40635_2024_632_MOESM2_ESM.pdf]

Homer Known Motif Enrichment Results

Total Target Sequences = 2079, Total Background Sequences = 47382

Rank	Motif	Name	P-value	log P-pvalue	q-value (Benjamini)	# Target Sequences with Motif	% of Targets Sequences with Motif	# Background Sequences with Motif	% of Background Sequences with Motif	Motif File	SVG
1	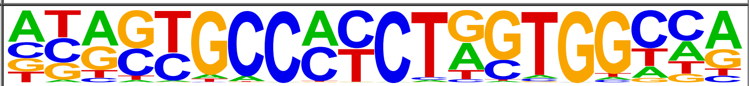	CTCF(Zf)/CD4+-CTCF-ChIP-Seq(Barski_et_al.)/Homer	1e-112	-2.597e+02	0.0000	314.0	15.10%	1511.5	3.19%	motif file (matrix)	svg
2	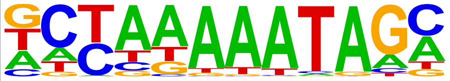	Mef2c(MADS)/GM12878-Mef2c-ChIP-Seq(GSE32465)/Homer	1e-94	-2.179e+02	0.0000	513.0	24.66%	4343.2	9.17%	motif file (matrix)	svg
3	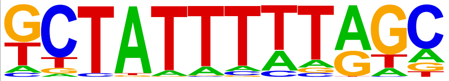	Mef2d(MADS)/Retina-Mef2d-ChIP-Seq(GSE61391)/Homer	1e-93	-2.147e+02	0.0000	320.0	15.38%	1876.2	3.96%	motif file (matrix)	svg
4	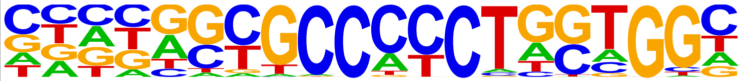	BORIS(Zf)/K562-CTCFL-ChIP-Seq(GSE32465)/Homer	1e-77	-1.786e+02	0.0000	309.0	14.86%	2044.6	4.32%	motif file (matrix)	svg
5	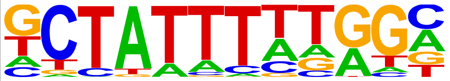	Mef2b(MADS)/HEK293-Mef2b.V5-ChIP-Seq(GSE67450)/Homer	1e-75	-1.749e+02	0.0000	737.0	35.43%	8655.7	18.28%	motif file (matrix)	svg
6	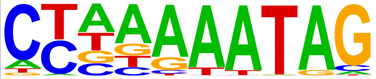	Mef2a(MADS)/HL1-Mef2a.biotin-ChIP-Seq(GSE21529)/Homer	1e-73	-1.688e+02	0.0000	471.0	22.64%	4371.6	9.23%	motif file (matrix)	svg
7	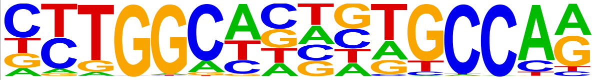	NF1(CTF)/LNCAP-NF1-ChIP-Seq(Unpublished)/Homer	1e-52	-1.207e+02	0.0000	463.0	22.26%	5022.8	10.61%	motif file (matrix)	svg
8	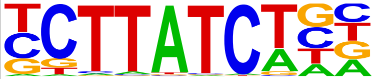	Gata6(Zf)/HUG1N-GATA6-ChIP-Seq(GSE51936)/Homer	1e-44	-1.035e+02	0.0000	747.0	35.91%	10530.9	22.24%	motif file (matrix)	svg
9	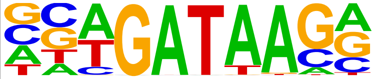	Gata4(Zf)/Heart-Gata4-ChIP-Seq(GSE35151)/Homer	1e-42	-9.899e+01	0.0000	797.0	38.32%	11643.7	24.59%	motif file (matrix)	svg
10	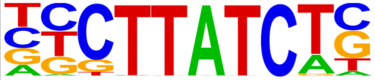	Gata2(Zf)/K562-GATA2-ChIP-Seq(GSE18829)/Homer	1e-40	-9.435e+01	0.0000	588.0	28.27%	7781.2	16.43%	motif file (matrix)	svg
11	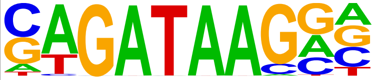	Gata1(Zf)/K562-GATA1-ChIP-Seq(GSE18829)/Homer	1e-38	-8.946e+01	0.0000	536.0	25.77%	6961.4	14.70%	motif file (matrix)	svg
12	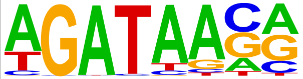	GATA3(Zf)/iTreg-Gata3-ChIP-Seq(GSE20898)/Homer	1e-32	-7.437e+01	0.0000	1001.0	48.12%	16740.6	35.36%	motif file (matrix)	svg

13	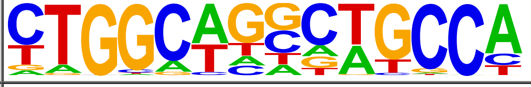	Tlx?(NR)/NPC-H3K4me1-ChIP-Seq(GSE16256)/Homer	1e-32	-7.399e+01	0.0000	461.0	22.16%	6016.7	12.71%	motif file (matrix)	svg
14	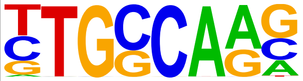	NF1-halfsite(CTF)/LNCaP-NF1-ChIP-Seq(Unpublished)/Homer	1e-26	-6.206e+01	0.0000	1176.0	56.54%	21143.4	44.65%	motif file (matrix)	svg
15	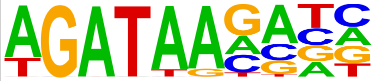	TRPS1(Zf)/MCF7-TRPS1-ChIP-Seq(GSE107013)/Homer	1e-25	-5.789e+01	0.0000	1190.0	57.21%	21660.8	45.75%	motif file (matrix)	svg
16	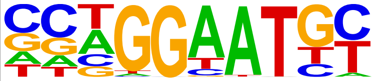	TEAD4(TEA)/Tropoblast-Tead4-ChIP-Seq(GSE37350)/Homer	1e-19	-4.413e+01	0.0000	616.0	29.62%	10011.9	21.15%	motif file (matrix)	svg
17	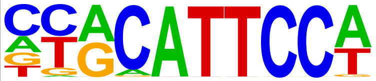	TEAD1(TEAD)/HepG2-TEAD1-ChIP-Seq(Encode)/Homer	1e-17	-4.143e+01	0.0000	664.0	31.92%	11113.5	23.47%	motif file (matrix)	svg
18	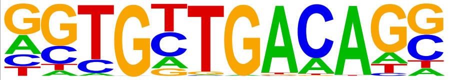	Tbx20(T-box)/Heart-Tbx20-ChIP-Seq(GSE29636)/Homer	1e-14	-3.410e+01	0.0000	235.0	11.30%	3119.5	6.59%	motif file (matrix)	svg
19	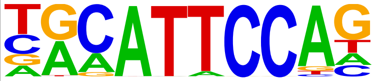	TEAD3(TEA)/HepG2-TEAD3-ChIP-Seq(Encode)/Homer	1e-14	-3.376e+01	0.0000	733.0	35.24%	12950.0	27.35%	motif file (matrix)	svg
20	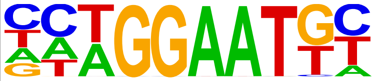	TEAD(TEA)/Fibroblast-PU.1-ChIP-Seq(Unpublished)/Homer	1e-14	-3.249e+01	0.0000	473.0	22.74%	7677.2	16.21%	motif file (matrix)	svg
21	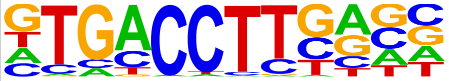	ERRg(NR)/Kidney-ESRRG-ChIP-Seq(GSE104905)/Homer	1e-12	-2.823e+01	0.0000	608.0	29.23%	10648.6	22.49%	motif file (matrix)	svg
22	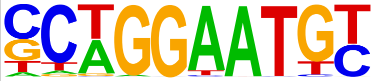	TEAD2(TEA)/Py2T-Tead2-ChIP-Seq(GSE55709)/Homer	1e-11	-2.577e+01	0.0000	388.0	18.65%	6314.8	13.34%	motif file (matrix)	svg
23	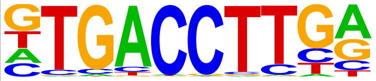	Esrrb(NR)/mES-Esrrb-ChIP-Seq(GSE11431)/Homer	1e-10	-2.394e+01	0.0000	488.0	23.46%	8422.8	17.79%	motif file (matrix)	svg
24	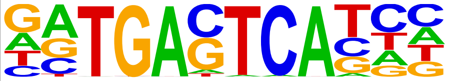	Jun-AP1(bZIP)/K562-cJun-ChIP-Seq(GSE31477)/Homer	1e-8	-2.058e+01	0.0000	185.0	8.89%	2662.0	5.62%	motif file (matrix)	svg
25	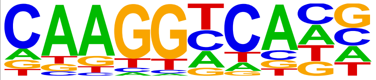	SF1(NR)/H295R-Nr5a1-ChIP-Seq(GSE44220)/Homer	1e-8	-1.952e+01	0.0000	368.0	17.69%	6242.3	13.18%	motif file (matrix)	svg
26	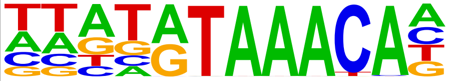	Foxf1(Forkhead)/Lung-Foxf1-ChIP-Seq(GSE77951)/Homer	1e-8	-1.936e+01	0.0000	537.0	25.82%	9724.8	20.54%	motif file (matrix)	svg
27	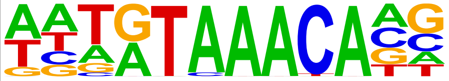	FoxL2(Forkhead)/Ovary-FoxL2-ChIP-Seq(GSE60858)/Homer	1e-8	-1.928e+01	0.0000	507.0	24.38%	9103.7	19.23%	motif file (matrix)	svg
28	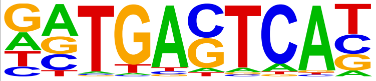	JunB(bZIP)/DendriticCells-Junb-ChIP-Seq(GSE36099)/Homer	1e-8	-1.851e+01	0.0000	362.0	17.40%	6179.9	13.05%	motif file (matrix)	svg

29	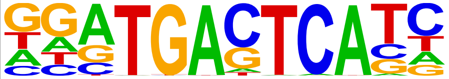	Fra2(bZIP)/Striatum-Fra2-ChIP-Seq(GSE43429)/Homer	1e-8	-1.844e+01	0.0000	320.0	15.38%	5343.1	11.28%	motif file (matrix)	svg
30	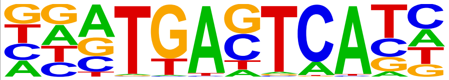	Fra1(bZIP)/BT549-Fra1-ChIP-Seq(GSE46166)/Homer	1e-7	-1.821e+01	0.0000	356.0	17.12%	6076.6	12.83%	motif file (matrix)	svg
31	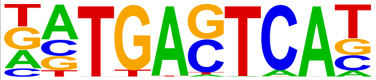	BATF(bZIP)/Th17-BATF-ChIP-Seq(GSE39756)/Homer	1e-7	-1.815e+01	0.0000	413.0	19.86%	7236.2	15.28%	motif file (matrix)	svg
32	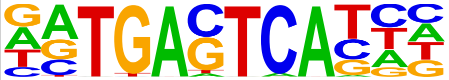	Atf3(bZIP)/GBM-ATF3-ChIP-Seq(GSE33912)/Homer	1e-7	-1.813e+01	0.0000	415.0	19.95%	7278.9	15.37%	motif file (matrix)	svg
33	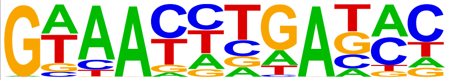	Six2(Homeobox)/NephronProgenitor-Six2-ChIP-Seq(GSE39837)/Homer	1e-7	-1.774e+01	0.0000	608.0	29.23%	11341.3	23.95%	motif file (matrix)	svg
34	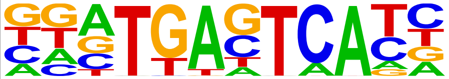	Fos(bZIP)/TSC-Fos-ChIP-Seq(GSE110950)/Homer	1e-7	-1.766e+01	0.0000	374.0	17.98%	6472.4	13.67%	motif file (matrix)	svg
35	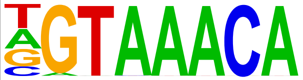	Foxo3(Forkhead)/U2OS-Foxo3-ChIP-Seq(E-MTAB-2701)/Homer	1e-7	-1.675e+01	0.0000	469.0	22.55%	8485.1	17.92%	motif file (matrix)	svg
36	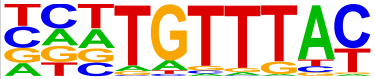	FOXK1(Forkhead)/HEK293-FOXK1-ChIP-Seq(GSE51673)/Homer	1e-7	-1.625e+01	0.0000	615.0	29.57%	11607.4	24.51%	motif file (matrix)	svg
37	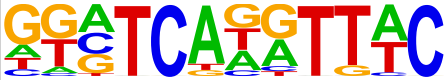	Six1(Homeobox)/Myoblast-Six1-ChIP-Chip(GSE20150)/Homer	1e-6	-1.589e+01	0.0000	188.0	9.04%	2903.8	6.13%	motif file (matrix)	svg
38	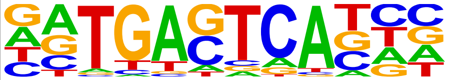	Fosl2(bZIP)/3T3L1-Fosl2-ChIP-Seq(GSE56872)/Homer	1e-6	-1.582e+01	0.0000	234.0	11.25%	3789.3	8.00%	motif file (matrix)	svg
39	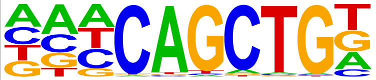	Ap4(bHLH)/AML-Tfap4-ChIP-Seq(GSE45738)/Homer	1e-6	-1.555e+01	0.0000	818.0	39.33%	16078.3	33.96%	motif file (matrix)	svg
40	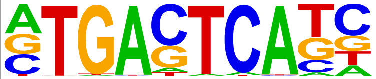	AP-1(bZIP)/ThioMac-PU.1-ChIP-Seq(GSE21512)/Homer	1e-6	-1.543e+01	0.0000	460.0	22.12%	8392.7	17.73%	motif file (matrix)	svg
41	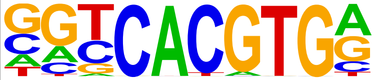	USF1(bHLH)/GM12878-Usf1-ChIP-Seq(GSE32465)/Homer	1e-6	-1.535e+01	0.0000	315.0	15.14%	5420.4	11.45%	motif file (matrix)	svg
42	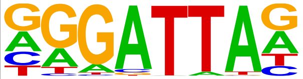	GSC(Homeobox)/FrogEmbryos-GSC-ChIP-Seq(DRA000576)/Homer	1e-6	-1.510e+01	0.0000	621.0	29.86%	11831.2	24.99%	motif file (matrix)	svg
43	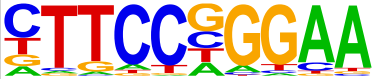	Stat3(Stat)/mES-Stat3-ChIP-Seq(GSE11431)/Homer	1e-6	-1.469e+01	0.0000	358.0	17.21%	6335.7	13.38%	motif file (matrix)	svg
44	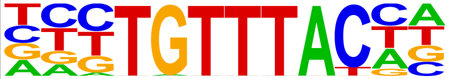	FOXP1(Forkhead)/H9-FOXP1-ChIP-Seq(GSE31006)/Homer	1e-6	-1.453e+01	0.0000	290.0	13.94%	4967.9	10.49%	motif file (matrix)	svg

45	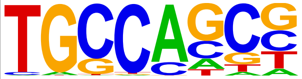	HIC1(Zf)/Treg-ZBTB29-ChIP-Seq(GSE99889)/Homer	1e-6	-1.429e+01	0.0000	1154.0	55.48%	23743.2	50.15%	motif file (matrix)	svg
46	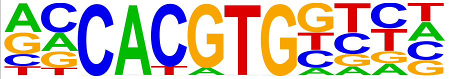	Max(bHLH)/K562-Max-ChIP-Seq(GSE31477)/Homer	1e-6	-1.403e+01	0.0000	430.0	20.67%	7870.2	16.62%	motif file (matrix)	svg
47	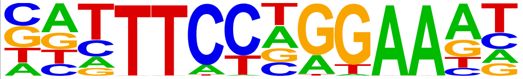	STAT1(Stat)/HelaS3-STAT1-ChIP-Seq(GSE12782)/Homer	1e-5	-1.297e+01	0.0000	223.0	10.72%	3724.2	7.87%	motif file (matrix)	svg
48	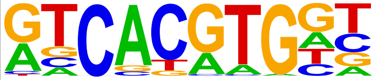	Usf2(bHLH)/C2C12-Usf2-ChIP-Seq(GSE36030)/Homer	1e-5	-1.292e+01	0.0000	222.0	10.67%	3707.8	7.83%	motif file (matrix)	svg
49	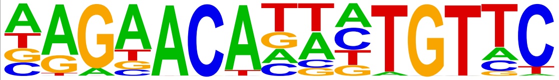	PGR(NR)/EndoStromal-PGR-ChIP-Seq(GSE69539)/Homer	1e-5	-1.279e+01	0.0000	177.0	8.51%	2837.0	5.99%	motif file (matrix)	svg
50	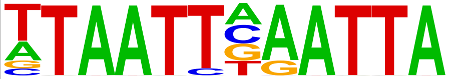	Phox2b(Homeobox)/CLBGA-PHOX2B-ChIP-Seq(GSE90683)/Homer	1e-5	-1.269e+01	0.0000	116.0	5.58%	1693.1	3.58%	motif file (matrix)	svg
51	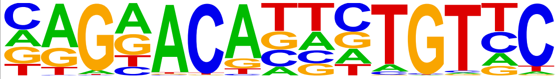	GRE(NR),IR3/RAW264.7-GRE-ChIP-Seq(Unpublished)/Homer	1e-5	-1.267e+01	0.0000	188.0	9.04%	3055.5	6.45%	motif file (matrix)	svg
52	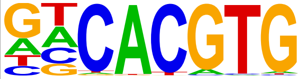	CLOCK(bHLH)/Liver-Clock-ChIP-Seq(GSE39860)/Homer	1e-5	-1.256e+01	0.0000	366.0	17.60%	6649.5	14.04%	motif file (matrix)	svg
53	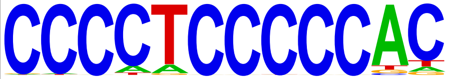	Zfp281(Zf)/ES-Zfp281-ChIP-Seq(GSE81042)/Homer	1e-5	-1.252e+01	0.0000	195.0	9.38%	3199.2	6.76%	motif file (matrix)	svg
54	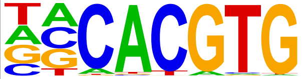	NPAS(bHLH)/Liver-NPAS-ChIP-Seq(GSE39860)/Homer	1e-5	-1.222e+01	0.0000	896.0	43.08%	18139.0	38.31%	motif file (matrix)	svg
55	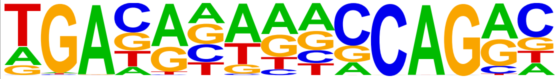	Hand2(bHLH)/Mesoderm-Hand2-ChIP-Seq(GSE61475)/Homer	1e-5	-1.220e+01	0.0000	388.0	18.65%	7134.2	15.07%	motif file (matrix)	svg
56	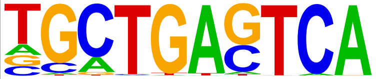	Bach2(bZIP)/OCILy7-Bach2-ChIP-Seq(GSE44420)/Homer	1e-5	-1.217e+01	0.0000	149.0	7.16%	2330.9	4.92%	motif file (matrix)	svg
57	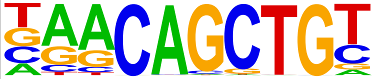	Myf5(bHLH)/GM-Myf5-ChIP-Seq(GSE24852)/Homer	1e-5	-1.208e+01	0.0000	520.0	25.00%	9932.1	20.98%	motif file (matrix)	svg
58	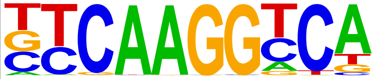	Nr5a2(NR)/mES-Nr5a2-ChIP-Seq(GSE19019)/Homer	1e-5	-1.206e+01	0.0000	385.0	18.51%	7082.3	14.96%	motif file (matrix)	svg
59	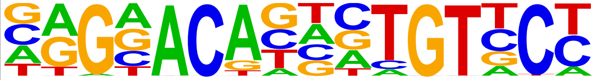	GRE(NR),IR3/A549-GR-ChIP-Seq(GSE32465)/Homer	1e-5	-1.197e+01	0.0000	117.0	5.62%	1739.7	3.67%	motif file (matrix)	svg
60	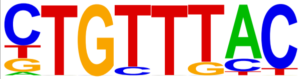	Foxo1(Forkhead)/RAW-Foxo1-ChIP-Seq(Fan_et_al.)/Homer	1e-5	-1.196e+01	0.0000	1061.0	51.01%	21878.7	46.21%	motif file (matrix)	svg

61	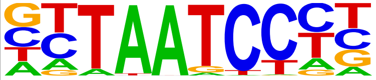	Otx2(Homeobox)/EpiLC-Otx2-ChIP-Seq(GSE56098)/Homer	1e-5	-1.168e+01	0.0001	432.0	20.77%	8099.4	17.11%	motif file (matrix)	svg
62	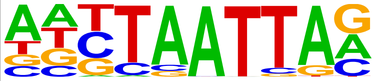	Lhx3(Homeobox)/Neuron-Lhx3-ChIP-Seq(GSE31456)/Homer	1e-4	-1.144e+01	0.0001	820.0	39.42%	16534.6	34.92%	motif file (matrix)	svg
63	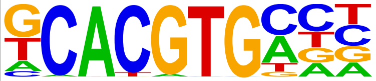	bHLHE40(bHLH)/HepG2-BHLHE40-ChIP-Seq(GSE31477)/Homer	1e-4	-1.122e+01	0.0001	203.0	9.76%	3428.3	7.24%	motif file (matrix)	svg
64	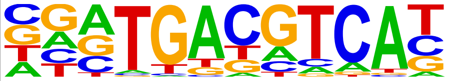	Atf2(bZIP)/3T3L1-Atf2-ChIP-Seq(GSE56872)/Homer	1e-4	-1.117e+01	0.0001	226.0	10.87%	3889.3	8.21%	motif file (matrix)	svg
65	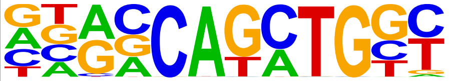	Atoh1(bHLH)/Cerebellum-Atoh1-ChIP-Seq(GSE22111)/Homer	1e-4	-1.106e+01	0.0001	733.0	35.24%	14654.3	30.95%	motif file (matrix)	svg
66	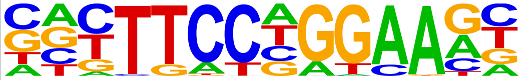	Stat3+il21(Stat)/CD4-Stat3-ChIP-Seq(GSE19198)/Homer	1e-4	-1.101e+01	0.0001	479.0	23.03%	9154.9	19.34%	motif file (matrix)	svg
67	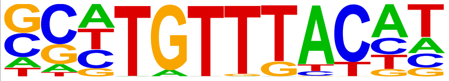	FOXK2(Forkhead)/U2OS-FOXK2-ChIP-Seq(E-MTAB-2204)/Homer	1e-4	-1.100e+01	0.0001	399.0	19.18%	7461.1	15.76%	motif file (matrix)	svg
68	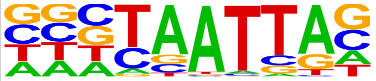	DLX2(Homeobox)/BasalGanglia-Dlx2-ChIP-seq(GSE124936)/Homer	1e-4	-1.100e+01	0.0001	761.0	36.59%	15278.7	32.27%	motif file (matrix)	svg
69	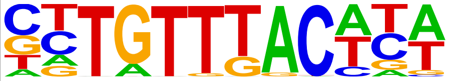	Foxa2(Forkhead)/Liver-Foxa2-ChIP-Seq(GSE25694)/Homer	1e-4	-1.099e+01	0.0001	488.0	23.46%	9348.6	19.74%	motif file (matrix)	svg
70	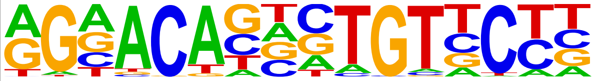	ARE(NR)/LNCAP-AR-ChIP-Seq(GSE27824)/Homer	1e-4	-1.079e+01	0.0001	206.0	9.90%	3513.5	7.42%	motif file (matrix)	svg
71	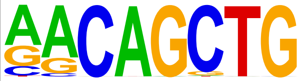	MyoG(bHLH)/C2C12-MyoG-ChIP-Seq(GSE36024)/Homer	1e-4	-1.073e+01	0.0001	717.0	34.47%	14338.3	30.28%	motif file (matrix)	svg
72	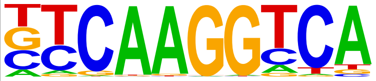	Nr5a2(NR)/Pancreas-LRH1-ChIP-Seq(GSE34295)/Homer	1e-4	-1.058e+01	0.0002	492.0	23.65%	9472.9	20.01%	motif file (matrix)	svg
73	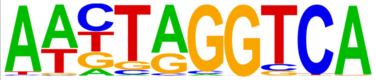	RORgt(NR)/EL4-RORgt.Flag-ChIP-Seq(GSE56019)/Homer	1e-4	-1.048e+01	0.0002	101.0	4.86%	1504.0	3.18%	motif file (matrix)	svg
74	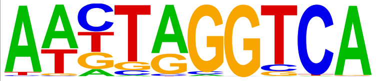	RORgt(NR)/EL4-RORgt.Flag-ChIP-Seq(GSE56019)/Homer	1e-4	-1.048e+01	0.0002	101.0	4.86%	1504.0	3.18%	motif file (matrix)	svg
75	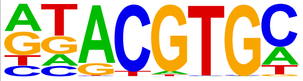	HIF-1b(HLH)/T47D-HIF1b-ChIP-Seq(GSE59937)/Homer	1e-4	-1.045e+01	0.0002	603.0	28.99%	11878.7	25.09%	motif file (matrix)	svg
76	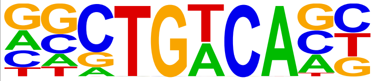	Meis1(Homeobox)/MastCells-Meis1-ChIP-Seq(GSE48085)/Homer	1e-4	-1.029e+01	0.0002	1036.0	49.81%	21508.9	45.43%	motif file (matrix)	svg

77	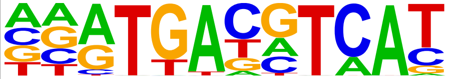	CREB5(bZIP)/LNCaP-CREB5.V5-ChIP-Seq(GSE137775)/Homer	1e-4	-1.020e+01	0.0002	243.0	11.68%	4295.5	9.07%	motif file (matrix)	svg
78	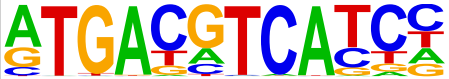	c-Jun-CRE(bZIP)/K562-cJun-ChIP-Seq(GSE31477)/Homer	1e-4	-1.011e+01	0.0002	208.0	10.00%	3595.7	7.59%	motif file (matrix)	svg
79	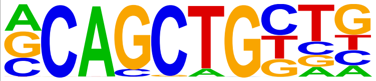	Tcf12(bHLH)/GM12878-Tcf12-ChIP-Seq(GSE32465)/Homer	1e-4	-1.008e+01	0.0002	656.0	31.54%	13073.9	27.61%	motif file (matrix)	svg
80	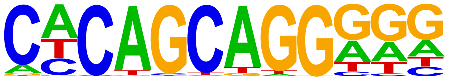	Unknown-ESC-element(?)/mES-Nanog-ChIP-Seq(GSE11724)/Homer	1e-4	-1.006e+01	0.0002	442.0	21.25%	8454.2	17.86%	motif file (matrix)	svg
81	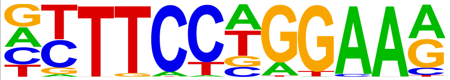	STAT4(Stat)/CD4-Stat4-ChIP-Seq(GSE22104)/Homer	1e-4	-1.003e+01	0.0002	601.0	28.89%	11879.8	25.09%	motif file (matrix)	svg
82	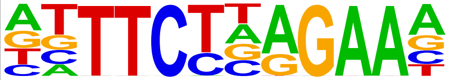	STAT5(Stat)/mCD4+-Stat5-ChIP-Seq(GSE12346)/Homer	1e-4	-9.814e+00	0.0003	244.0	11.73%	4342.1	9.17%	motif file (matrix)	svg
83	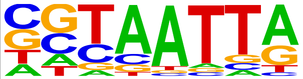	DLX5(Homeobox)/BasalGanglia-Dlx5-ChIP-seq(GSE124936)/Homer	1e-4	-9.814e+00	0.0003	421.0	20.24%	8031.9	16.96%	motif file (matrix)	svg
84	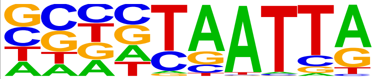	DLX1(Homeobox)/BasalGanglia-Dlx1-ChIP-seq(GSE124936)/Homer	1e-4	-9.806e+00	0.0003	678.0	32.60%	13585.5	28.69%	motif file (matrix)	svg
85	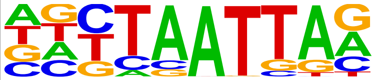	Lhx1(Homeobox)/EmbryoCarcinoma-Lhx1-ChIP-Seq(GSE70957)/Homer	1e-4	-9.788e+00	0.0003	575.0	27.64%	11340.6	23.95%	motif file (matrix)	svg
86	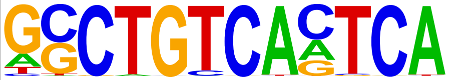	PBX1(Homeobox)/MCF7-PBX1-ChIP-Seq(GSE28007)/Homer	1e-4	-9.696e+00	0.0003	85.0	4.09%	1240.7	2.62%	motif file (matrix)	svg
87	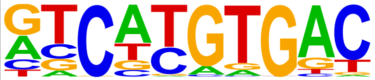	MITF(bHLH)/MastCells-MITF-ChIP-Seq(GSE48085)/Homer	1e-4	-9.613e+00	0.0003	588.0	28.27%	11641.4	24.59%	motif file (matrix)	svg
88	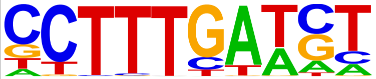	LEF1(HMG)/H1-LEF1-ChIP-Seq(GSE64758)/Homer	1e-4	-9.594e+00	0.0003	434.0	20.87%	8328.1	17.59%	motif file (matrix)	svg
89	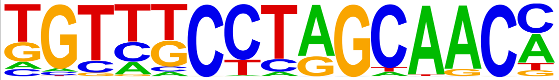	Rfx6(HTH)/Min6b1-Rfx6.HA-ChIP-Seq(GSE62844)/Homer	1e-4	-9.461e+00	0.0004	698.0	33.56%	14064.1	29.70%	motif file (matrix)	svg
90	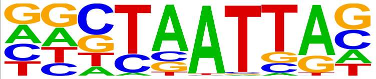	En1(Homeobox)/SUM149-EN1-ChIP-Seq(GSE120957)/Homer	1e-4	-9.393e+00	0.0004	898.0	43.17%	18513.4	39.10%	motif file (matrix)	svg
91	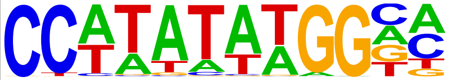	CArG(MADS)/PUER-Srf-ChIP-Seq(Sullivan_et_al.)/Homer	1e-4	-9.250e+00	0.0005	203.0	9.76%	3550.9	7.50%	motif file (matrix)	svg
92	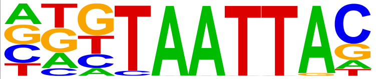	Dlx3(Homeobox)/Kerainocytes-Dlx3-ChIP-Seq(GSE89884)/Homer	1e-3	-9.180e+00	0.0005	361.0	17.36%	6820.2	14.40%	motif file (matrix)	svg

93	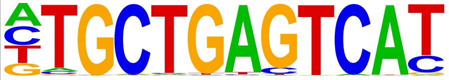	Nrf2(bZIP)/Lymphoblast-Nrf2-ChIP-Seq(GSE37589)/Homer	1e-3	-9.075e+00	0.0005	41.0	1.97%	491.2	1.04%	motif file (matrix)	svg
94	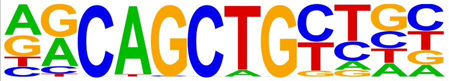	MyoD(bHLH)/Myotube-MyoD-ChIP-Seq(GSE21614)/Homer	1e-3	-9.014e+00	0.0006	539.0	25.91%	10643.4	22.48%	motif file (matrix)	svg
95	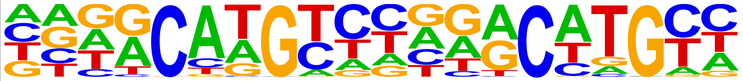	p63(p53)/Keratinocyte-p63-ChIP-Seq(GSE17611)/Homer	1e-3	-8.786e+00	0.0007	253.0	12.16%	4602.3	9.72%	motif file (matrix)	svg
96	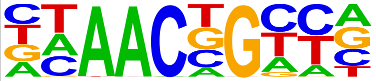	BMYB(HTH)/Hela-BMYB-ChIP-Seq(GSE27030)/Homer	1e-3	-8.689e+00	0.0008	863.0	41.49%	17820.1	37.64%	motif file (matrix)	svg
97	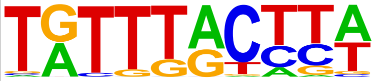	FOXM1(Forkhead)/MCF7-FOXM1-ChIP-Seq(GSE72977)/Homer	1e-3	-8.585e+00	0.0008	575.0	27.64%	11473.7	24.23%	motif file (matrix)	svg
98	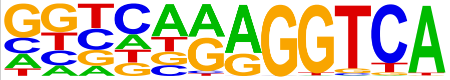	COUP-TFII(NR)/K562-NR2F1-ChIP-Seq(Encode)/Homer	1e-3	-8.520e+00	0.0009	842.0	40.48%	17373.6	36.69%	motif file (matrix)	svg
99	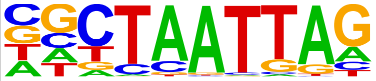	LHX9(Homeobox)/Hct116-LHX9.V5-ChIP-Seq(GSE116822)/Homer	1e-3	-8.466e+00	0.0009	732.0	35.19%	14935.4	31.54%	motif file (matrix)	svg
100	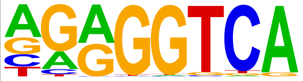	COUP-TFII(NR)/Artia-Nr2f2-ChIP-Seq(GSE46497)/Homer	1e-3	-8.453e+00	0.0009	951.0	45.72%	19826.6	41.87%	motif file (matrix)	svg
101	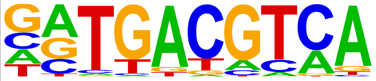	Atf1(bZIP)/K562-ATF1-ChIP-Seq(GSE31477)/Homer	1e-3	-8.394e+00	0.0010	397.0	19.09%	7658.0	16.17%	motif file (matrix)	svg
102	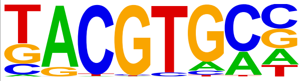	HIF-1a(bHLH)/MCF7-HIF1a-ChIP-Seq(GSE28352)/Homer	1e-3	-8.374e+00	0.0010	158.0	7.60%	2705.6	5.71%	motif file (matrix)	svg
103	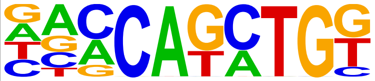	BHLHA15(bHLH)/NIH3T3-BHLHB8.HA-ChIP-Seq(GSE119782)/Homer	1e-3	-8.277e+00	0.0011	867.0	41.68%	17964.9	37.94%	motif file (matrix)	svg
104	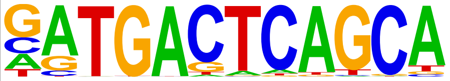	NF-E2(bZIP)/K562-NFE2-ChIP-Seq(GSE31477)/Homer	1e-3	-8.251e+00	0.0011	46.0	2.21%	596.1	1.26%	motif file (matrix)	svg
105	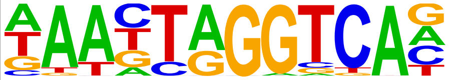	RORg(NR)/Liver-Rorc-ChIP-Seq(GSE101115)/Homer	1e-3	-8.046e+00	0.0013	71.0	3.41%	1048.0	2.21%	motif file (matrix)	svg
106	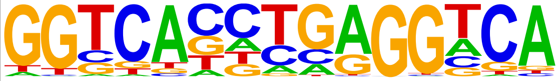	THRb(NR)/HepG2-THRb.Flag-ChIP-Seq(Encode)/Homer	1e-3	-8.029e+00	0.0014	385.0	18.51%	7438.3	15.71%	motif file (matrix)	svg
107	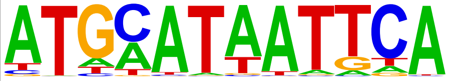	Pit1+1bp(Homeobox)/GCrat-Pit1-ChIP-Seq(GSE58009)/Homer	1e-3	-8.012e+00	0.0014	179.0	8.61%	3149.2	6.65%	motif file (matrix)	svg
108	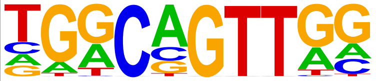	AMYB(HTH)/Testes-AMYB-ChIP-Seq(GSE44588)/Homer	1e-3	-7.965e+00	0.0014	876.0	42.12%	18208.5	38.46%	motif file (matrix)	svg

109	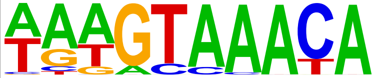	FOXA1(Forkhead)/LNCAP-FOXA1-ChIP-Seq(GSE27824)/Homer	1e-3	-7.938e+00	0.0014	647.0	31.11%	13127.8	27.73%	motif file (matrix)	svg
110	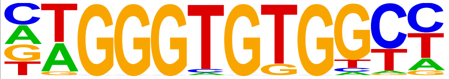	EKLF(Zf)/Erythrocyte-Klf1-ChIP-Seq(GSE20478)/Homer	1e-3	-7.894e+00	0.0015	158.0	7.60%	2735.2	5.78%	motif file (matrix)	svg
111	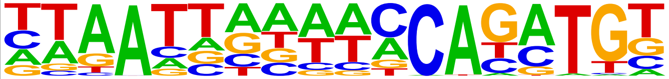	Pitx1:Ebox(Homeobox,bHLH)/Hindlimb-Pitx1-ChIP-Seq(GSE41591)/Homer	1e-3	-7.842e+00	0.0016	118.0	5.67%	1948.9	4.12%	motif file (matrix)	svg
112	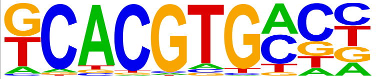	bHLHE41(bHLH)/proB-Bhlhe41-ChIP-Seq(GSE93764)/Homer	1e-3	-7.800e+00	0.0016	610.0	29.33%	12332.5	26.05%	motif file (matrix)	svg
113	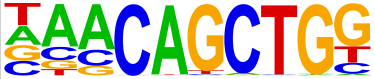	Tcf21(bHLH)/ArterySmoothMuscle-Tcf21-ChIP-Seq(GSE61369)/Homer	1e-3	-7.742e+00	0.0017	636.0	30.58%	12911.0	27.27%	motif file (matrix)	svg
114	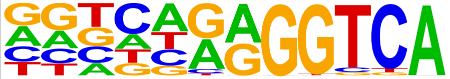	EAR2(NR)/K562-NR2F6-ChIP-Seq(Encode)/Homer	1e-3	-7.731e+00	0.0017	782.0	37.60%	16141.1	34.09%	motif file (matrix)	svg
115	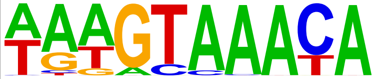	FOXA1(Forkhead)/MCF7-FOXA1-ChIP-Seq(GSE26831)/Homer	1e-3	-7.712e+00	0.0017	533.0	25.62%	10661.2	22.52%	motif file (matrix)	svg
116	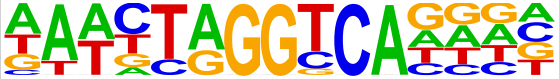	RORa(NR)/Liver-Rora-ChIP-Seq(GSE101115)/Homer	1e-3	-7.649e+00	0.0018	101.0	4.86%	1629.0	3.44%	motif file (matrix)	svg
117	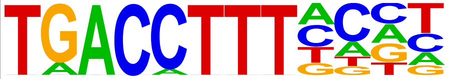	Nur77(NR)/K562-NR4A1-ChIP-Seq(GSE31363)/Homer	1e-3	-7.563e+00	0.0020	130.0	6.25%	2199.9	4.65%	motif file (matrix)	svg
118	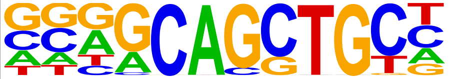	Ascl2(bHLH)/ESC-Ascl2-ChIP-Seq(GSE97712)/Homer	1e-3	-7.541e+00	0.0020	713.0	34.28%	14635.8	30.91%	motif file (matrix)	svg
119	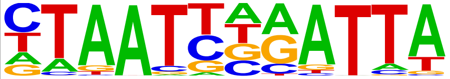	Phox2a(Homeobox)/Neuron-Phox2a-ChIP-Seq(GSE31456)/Homer	1e-3	-7.418e+00	0.0022	192.0	9.23%	3455.4	7.30%	motif file (matrix)	svg
120	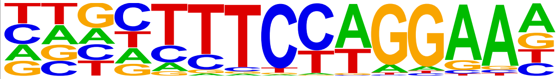	Bcl6(Zf)/Liver-Bcl6-ChIP-Seq(GSE31578)/Homer	1e-3	-7.280e+00	0.0025	804.0	38.65%	16694.2	35.26%	motif file (matrix)	svg
121	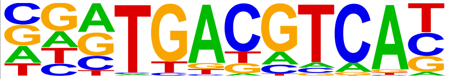	Atf7(bZIP)/3T3L1-Atf7-ChIP-Seq(GSE56872)/Homer	1e-3	-7.277e+00	0.0025	287.0	13.80%	5437.4	11.48%	motif file (matrix)	svg
122	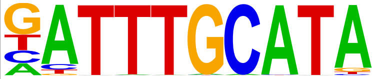	Oct11(POU,Homeobox)/NCIH1048-POU2F3-ChIP-seq(GSE115123)/Homer	1e-3	-7.155e+00	0.0028	175.0	8.41%	3128.7	6.61%	motif file (matrix)	svg
123	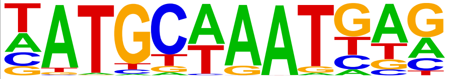	Oct6(POU,Homeobox)/NPC-Pou3f1-ChIP-Seq(GSE35496)/Homer	1e-3	-7.152e+00	0.0028	223.0	10.72%	4113.3	8.69%	motif file (matrix)	svg
124	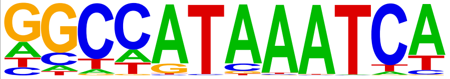	Hoxc9(Homeobox)/Ainv15-Hoxc9-ChIP-Seq(GSE21812)/Homer	1e-3	-7.104e+00	0.0029	277.0	13.32%	5243.4	11.07%	motif file (matrix)	svg

125	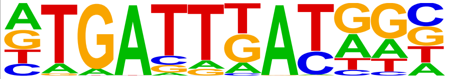	PBX2(Homeobox)/K562-PBX2-ChIP-Seq(Encode)/Homer	1e-3	-7.019e+00	0.0032	453.0	21.78%	9008.9	19.03%	motif file (matrix)	svg
126	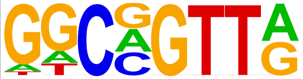	MYB(HTH)/ERMYB-Myb-ChIPSeq(GSE22095)/Homer	1e-3	-7.000e+00	0.0032	947.0	45.53%	19943.2	42.12%	motif file (matrix)	svg
127	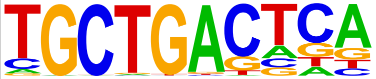	MafA(bZIP)/Islet-MafA-ChIP-Seq(GSE30298)/Homer	1e-3	-6.986e+00	0.0032	546.0	26.25%	11034.6	23.31%	motif file (matrix)	svg
128	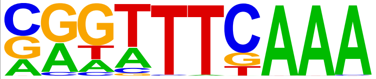	CHR(?)/Hela-CellCycle-Expression/Homer	1e-3	-6.950e+00	0.0033	349.0	16.78%	6782.4	14.32%	motif file (matrix)	svg
129	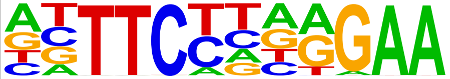	STAT6(Stat)/CD4-Stat6-ChIP-Seq(GSE22104)/Homer	1e-3	-6.931e+00	0.0033	339.0	16.30%	6571.9	13.88%	motif file (matrix)	svg
130	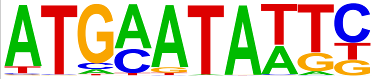	Brn2(POU,Homeobox)/NPC-Brn2-ChIP-Seq(GSE35496)/Homer	1e-2	-6.890e+00	0.0034	60.0	2.88%	890.8	1.88%	motif file (matrix)	svg
131	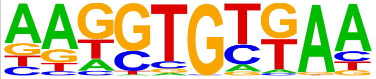	Tbr1(T-box)/Cortex-Tbr1-ChIP-Seq(GSE71384)/Homer	1e-2	-6.808e+00	0.0037	781.0	37.55%	16249.9	34.32%	motif file (matrix)	svg
132	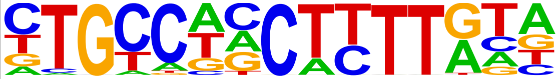	ZNF7(Zf)/HepG2-ZNF7.Flag-ChIP-Seq(Encode)/Homer	1e-2	-6.807e+00	0.0037	339.0	16.30%	6584.5	13.91%	motif file (matrix)	svg
133	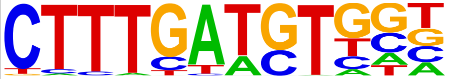	Tcf7(HMG)/GM12878-TCF7-ChIP-Seq(Encode)/Homer	1e-2	-6.806e+00	0.0037	223.0	10.72%	4142.8	8.75%	motif file (matrix)	svg
134	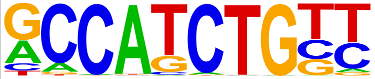	NeuroD1(bHLH)/Islet-NeuroD1-ChIP-Seq(GSE30298)/Homer	1e-2	-6.642e+00	0.0043	551.0	26.49%	11189.0	23.63%	motif file (matrix)	svg
135	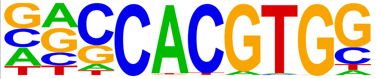	n-Myc(bHLH)/mES-nMyc-ChIP-Seq(GSE11431)/Homer	1e-2	-6.571e+00	0.0046	406.0	19.52%	8046.5	16.99%	motif file (matrix)	svg
136	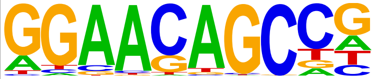	ZNF341(Zf)/EBV-ZNF341-ChIP-Seq(GSE113194)/Homer	1e-2	-6.463e+00	0.0050	471.0	22.64%	9466.9	19.99%	motif file (matrix)	svg
137	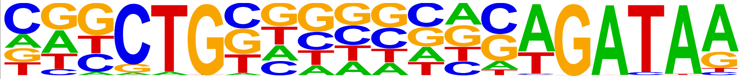	GATA:SCL(Zf,bHLH)/Ter119-SCL-ChIP-Seq(GSE18720)/Homer	1e-2	-6.458e+00	0.0050	97.0	4.66%	1614.8	3.41%	motif file (matrix)	svg
138	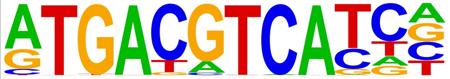	JunD(bZIP)/K562-JunD-ChIP-Seq/Homer	1e-2	-6.406e+00	0.0053	69.0	3.32%	1078.5	2.28%	motif file (matrix)	svg
139	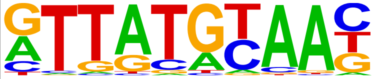	HLF(bZIP)/HSC-HLF.Flag-ChIP-Seq(GSE69817)/Homer	1e-2	-6.388e+00	0.0053	425.0	20.43%	8478.4	17.91%	motif file (matrix)	svg
140	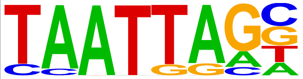	Lhx2(Homeobox)/HFSC-Lhx2-ChIP-Seq(GSE48068)/Homer	1e-2	-6.354e+00	0.0055	546.0	26.25%	11117.5	23.48%	motif file (matrix)	svg

141	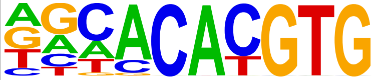	MNT(bHLH)/HepG2-MNT-ChIP-Seq(Encode)/Homer	1e-2	-6.263e+00	0.0059	598.0	28.75%	12272.9	25.92%	motif file (matrix)	svg
142	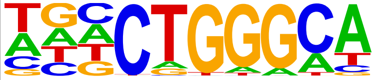	ZNF416(Zf)/HEK293-ZNF416.GFP-ChIP-Seq(GSE58341)/Homer	1e-2	-6.121e+00	0.0068	820.0	39.42%	17225.1	36.38%	motif file (matrix)	svg
143	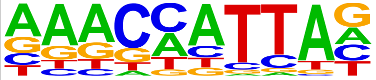	Barx1(Homeobox)/Stomach-Barx1.3xFlag-ChIP-Seq(GSE69483)/Homer	1e-2	-6.090e+00	0.0070	279.0	13.41%	5384.2	11.37%	motif file (matrix)	svg
144	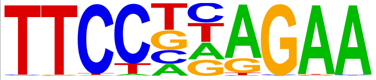	STAT6(Stat)/Macrophage-Stat6-ChIP-Seq(GSE38377)/Homer	1e-2	-6.061e+00	0.0071	343.0	16.49%	6751.2	14.26%	motif file (matrix)	svg
145	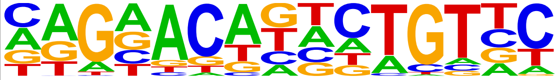	PR(NR)/T47D-PR-ChIP-Seq(GSE31130)/Homer	1e-2	-6.051e+00	0.0071	1168.0	56.15%	25114.3	53.04%	motif file (matrix)	svg
146	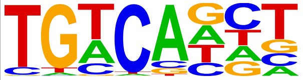	Tgif2(Homeobox)/mES-Tgif2-ChIP-Seq(GSE55404)/Homer	1e-2	-6.043e+00	0.0072	1531.0	73.61%	33518.6	70.79%	motif file (matrix)	svg
147	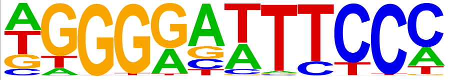	NFkB-p65(RHD)/GM12787-p65-ChIP-Seq(GSE19485)/Homer	1e-2	-5.989e+00	0.0075	320.0	15.38%	6267.9	13.24%	motif file (matrix)	svg
148	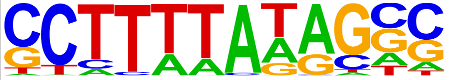	TATA-Box(TBP)/Promoter/Homer	1e-2	-5.965e+00	0.0076	719.0	34.57%	14995.1	31.67%	motif file (matrix)	svg
149	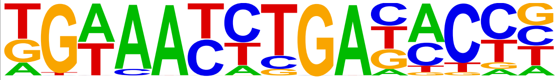	Six4(Homeobox)/MCF7-SIX4-ChIP-Seq(Encode)/Homer	1e-2	-5.827e+00	0.0087	38.0	1.83%	530.8	1.12%	motif file (matrix)	svg
150	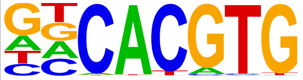	BMAL1(bHLH)/Liver-Bmal1-ChIP-Seq(GSE39860)/Homer	1e-2	-5.793e+00	0.0089	961.0	46.20%	20452.0	43.19%	motif file (matrix)	svg
151	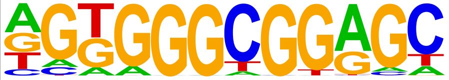	Sp5(Zf)/mES-Sp5.Flag-ChIP-Seq(GSE72989)/Homer	1e-2	-5.786e+00	0.0089	527.0	25.34%	10779.9	22.77%	motif file (matrix)	svg
152	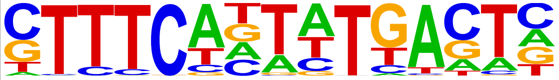	IRF:BATF(IRF:bZIP)/pDC-Irf8-ChIP-Seq(GSE66899)/Homer	1e-2	-5.742e+00	0.0093	81.0	3.89%	1341.0	2.83%	motif file (matrix)	svg
153	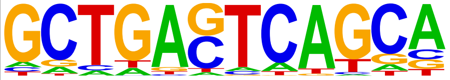	MafK(bZIP)/C2C12-MafK-ChIP-Seq(GSE36030)/Homer	1e-2	-5.659e+00	0.0100	158.0	7.60%	2894.3	6.11%	motif file (matrix)	svg
154	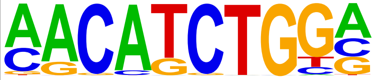	ZBTB18(Zf)/HEK293-ZBTB18.GFP-ChIP-Seq(GSE58341)/Homer	1e-2	-5.650e+00	0.0100	357.0	17.16%	7100.5	15.00%	motif file (matrix)	svg
155	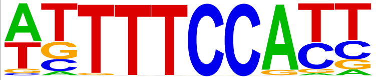	NFAT(RHD)/Jurkat-NFATC1-ChIP-Seq(Jolma_et_al.)/Homer	1e-2	-5.638e+00	0.0101	494.0	23.75%	10077.9	21.28%	motif file (matrix)	svg
156	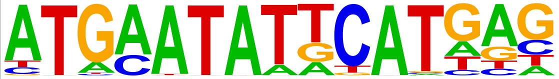	OCT:OCT(POU,Homeobox)/NPC-Brn1-ChIP-Seq(GSE35496)/Homer	1e-2	-5.604e+00	0.0104	10.0	0.48%	81.2	0.17%	motif file (matrix)	svg

157	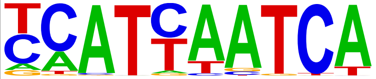	Pdx1(Homeobox)/Islet-Pdx1-ChIP-Seq(SRA008281)/Homer	1e-2	-5.469e+00	0.0118	504.0	24.23%	10320.0	21.80%	motif file (matrix)	svg
158	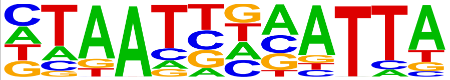	Prop1(Homeobox)/GHFT1-PROP1.biotin-ChIP-Seq(GSE77302)/Homer	1e-2	-5.458e+00	0.0119	288.0	13.85%	5643.6	11.92%	motif file (matrix)	svg
159	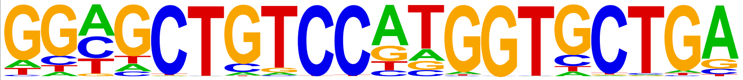	REST-NRSF(Zf)/Jurkat-NRSF-ChIP-Seq/Homer	1e-2	-5.414e+00	0.0123	12.0	0.58%	111.6	0.24%	motif file (matrix)	svg
160	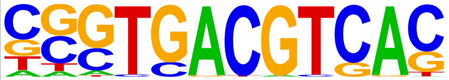	CRE(bZIP)/Promoter/Homer	1e-2	-5.408e+00	0.0123	143.0	6.88%	2606.1	5.50%	motif file (matrix)	svg
161	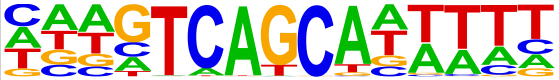	MafF(bZIP)/HepG2-MafF-ChIP-Seq(GSE31477)/Homer	1e-2	-5.364e+00	0.0128	158.0	7.60%	2918.2	6.16%	motif file (matrix)	svg
162	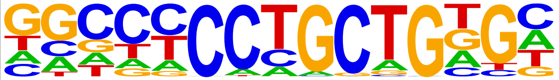	Zic3(Zf)/mES-Zic3-ChIP-Seq(GSE37889)/Homer	1e-2	-5.357e+00	0.0128	393.0	18.89%	7916.0	16.72%	motif file (matrix)	svg
163	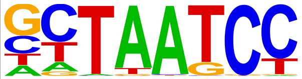	CRX(Homeobox)/Retina-Crx-ChIP-Seq(GSE20012)/Homer	1e-2	-5.294e+00	0.0135	1127.0	54.18%	24309.0	51.34%	motif file (matrix)	svg
164	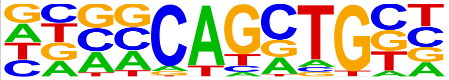	Ascl1(bHLH)/NeuralTubes-Ascl1-ChIP-Seq(GSE55840)/Homer	1e-2	-5.292e+00	0.0135	921.0	44.28%	19634.1	41.47%	motif file (matrix)	svg
165	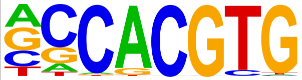	c-Myc(bHLH)/LNCAP-cMyc-ChIP-Seq(Unpublished)/Homer	1e-2	-5.283e+00	0.0135	247.0	11.88%	4790.6	10.12%	motif file (matrix)	svg
166	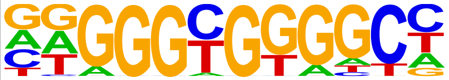	KLF1(Zf)/HUDEP2-KLF1-CutnRun(GSE136251)/Homer	1e-2	-5.267e+00	0.0137	493.0	23.70%	10108.3	21.35%	motif file (matrix)	svg
167	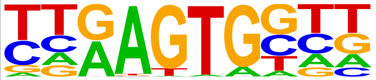	Bapx1(Homeobox)/VertebralCol-Bapx1-ChIP-Seq(GSE36672)/Homer	1e-2	-5.267e+00	0.0137	1116.0	53.65%	24062.3	50.82%	motif file (matrix)	svg
168	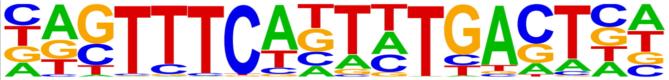	bZIP:IRF(bZIP,IRF)/Th17-BatF-ChIP-Seq(GSE39756)/Homer	1e-2	-5.232e+00	0.0140	250.0	12.02%	4860.0	10.26%	motif file (matrix)	svg
169	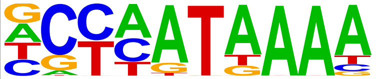	Hoxd13(Homeobox)/ChickenMSG-Hoxd13.Flag-ChIP-Seq(GSE86088)/Homer	1e-2	-5.162e+00	0.0149	786.0	37.79%	16622.2	35.11%	motif file (matrix)	svg
170	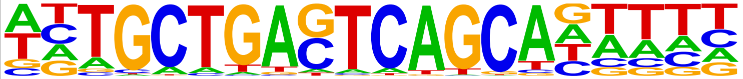	MafB(bZIP)/BMM-Mafb-ChIP-Seq(GSE75722)/Homer	1e-2	-5.161e+00	0.0149	272.0	13.08%	5335.3	11.27%	motif file (matrix)	svg
171	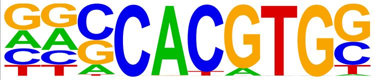	c-Myc(bHLH)/mES-cMyc-ChIP-Seq(GSE11431)/Homer	1e-2	-5.161e+00	0.0149	298.0	14.33%	5891.6	12.44%	motif file (matrix)	svg
172	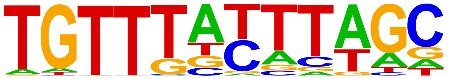	FoxD3(forkhead)/ZebrafishEmbryo-Foxd3.biotin-ChIP-seq(GSE106676)/Homer	1e-2	-5.131e+00	0.0151	481.0	23.12%	9865.7	20.84%	motif file (matrix)	svg

173	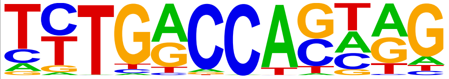	Bcl11a(Zf)/HSPC-BCL11A-ChIP-Seq(GSE104676)/Homer	1e-2	-5.119e+00	0.0152	468.0	22.50%	9582.2	20.24%	motif file (matrix)	svg
174	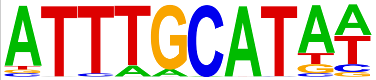	Oct4(POU,Homeobox)/mES-Oct4-ChIP-Seq(GSE11431)/Homer	1e-2	-5.104e+00	0.0154	237.0	11.39%	4597.7	9.71%	motif file (matrix)	svg
175	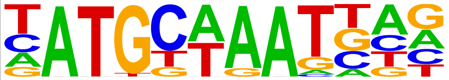	Brn1(POU,Homeobox)/NPC-Brn1-ChIP-Seq(GSE35496)/Homer	1e-2	-4.977e+00	0.0173	162.0	7.79%	3034.5	6.41%	motif file (matrix)	svg
176	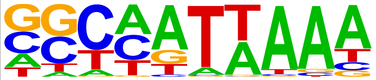	Unknown(Homeobox)/Limb-p300-ChIP-Seq/Homer	1e-2	-4.944e+00	0.0178	334.0	16.06%	6692.6	14.13%	motif file (matrix)	svg
177	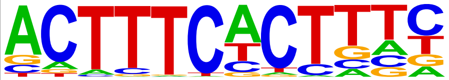	PRDM1(Zf)/Hela-PRDM1-ChIP-Seq(GSE31477)/Homer	1e-2	-4.906e+00	0.0184	324.0	15.58%	6481.0	13.69%	motif file (matrix)	svg
178	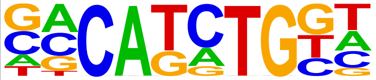	TCF4(bHLH)/SHSY5Y-TCF4-ChIP-Seq(GSE96915)/Homer	1e-2	-4.861e+00	0.0191	907.0	43.61%	19396.7	40.97%	motif file (matrix)	svg
179	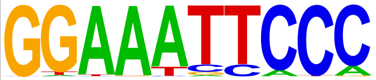	NFkB-p65-Rel(RHD)/ThioMac-LPS-Expression(GSE23622)/Homer	1e-2	-4.768e+00	0.0209	45.0	2.16%	698.7	1.48%	motif file (matrix)	svg
180	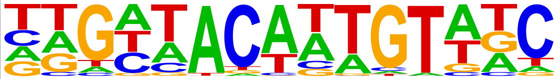	DMRT6(DM)/Testis-DMRT6-ChIP-Seq(GSE60440)/Homer	1e-2	-4.662e+00	0.0231	122.0	5.87%	2234.8	4.72%	motif file (matrix)	svg
181	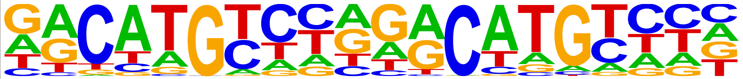	p53(p53)/Saos-p53-ChIP-Seq(GSE15780)/Homer	1e-2	-4.610e+00	0.0241	69.0	3.32%	1169.6	2.47%	motif file (matrix)	svg
182	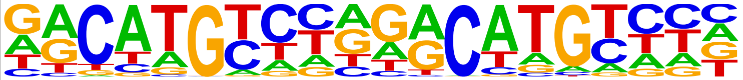	p53(p53)/Saos-p53-ChIP-Seq/Homer	1e-2	-4.610e+00	0.0241	69.0	3.32%	1169.6	2.47%	motif file (matrix)	svg
