# Supplementary material for: Catecholamine treatment induces reversible heart injury and cardiomyocyte gene expression
Source: Intensive Care Med Exp. 2024 May 11;12:48. doi: 10.1186/s40635-024-00632-9 (PMC11088585; doi:10.1186/s40635-024-00632-9)
Supplement: Supplementary file 3 — Supplementary Material 3. [file 40635_2024_632_MOESM3_ESM.docx]

**SUPPLEMENTAL INFORMATION**

**Catecholamine treatment induces reversible heart injury and cardiomyocyte gene expression**

**Running title: Catecholamine-induced heart injury**

Christine Bode^1^, Sebastian Preissl^1^, Lutz Hein^1,2^, Achim Lother^1,3^

^1^ Institute of Experimental and Clinical Pharmacology and Toxicology, Faculty of Medicine, University of Freiburg, Germany

^2^ BIOSS Centre for Biological Signaling Studies, University of Freiburg, Germany

^3^ Interdisciplinary Medical Intensive Care, Faculty of Medicine, University of Freiburg, Germany

**Suppl. Table S1: qRT-PCR primer sequences**

| **Gene symbol** | **Forward Primer** | **Reverse Primer** |
| --- | --- | --- |
| *Ctgf* | TGACCCCTGCGACCCACA | TACACCGACCCACCGAAGACACAG |
| *Fhl1* | ggaacacatcctgtgtgaggt | tcgaacttctccgacatggt |
| *Rps29* | ATGGGTCACCAGCAGCTCTA | AGCCTATGTCCTTCGCGTACT |

**Suppl. Figure S1. *Rps29* mRNA expression.** Ct values as determined by qPCR for Ribosomal protein S29 (*Rps29*) in heart tissue. n = 6 per group.

**
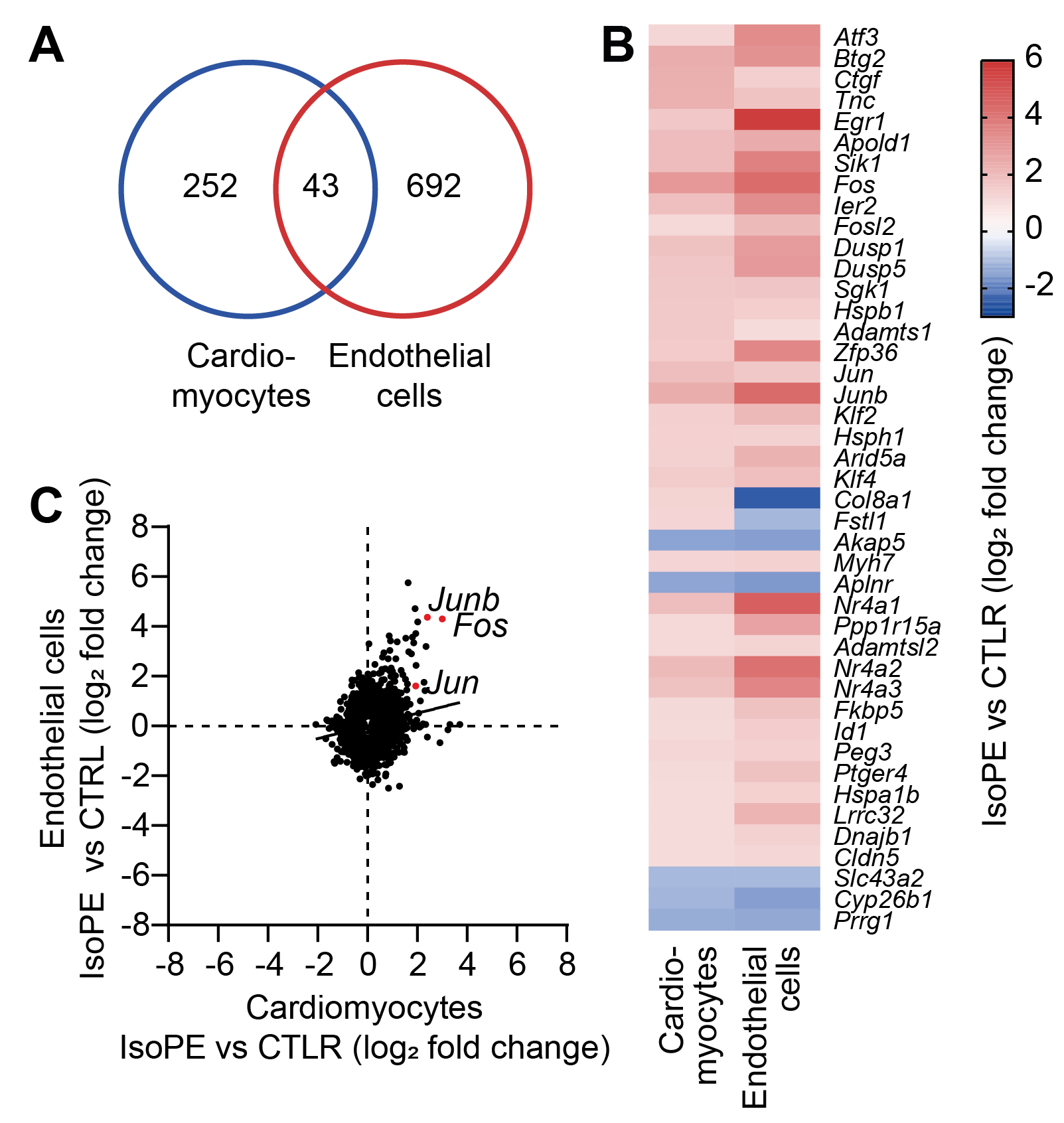
**

**Suppl. Figure S2: Gene expression response to adrenergic stimulation in cardiomyocytes compared to endothelial cells. A**, Venn diagram representing numbers of differentially expressed genes (fold change > 2 vs. CTRL, q < 0.05) after IsoPE treatment in cardiomyocytes, endothelial cells, or both. **B**, Heatmap representing the overlap of differentially expressed genes in cardiomyocytes and endothelial cells (fold change > 2 vs. CTRL, q < 0.05). **C**, Correlation of gene expression changes after IsoPE in cardiomyocytes vs. endothelial cells (q < 0.05). Gene expression in endothelial cells was derived from a previously published dataset [[20](#_ENREF_20)].
